# MUC1-C auto-regulatory complex with EBNA1 is responsible for latent Epstein-Barr virus-associated gastric cancer progression

**DOI:** 10.1038/s41388-025-03519-5

**Published:** 2025-08-05

**Authors:** Hiroki Ozawa, Yin Wang, Henry G. Withers, Naoki Haratake, Ayako Nakashoji, Atrayee Bhattacharya, Atsushi Fushimi, Chie Kikutake, Kazuhiro Yamanoi, Shaowen White, Keyi Wang, Tatsuaki Daimon, Keisuke Shigeta, Kazumasa Fukuda, Hirofumi Kawakubo, Yuko Kitagawa, Mark D. Long, Benjamin E. Gewurz, Donald Kufe

**Affiliations:** 1 Department of Medical Oncology, Dana-Farber Cancer Institute, Harvard Medical School, Boston, MA USA; 2 Department of Medicine, Brigham and Women’s Hospital, Harvard Medical School, Boston, MA USA; 3 Department of Biostatistics & Bioinformatics, Roswell Park Comprehensive Cancer Center, Buffalo, NY USA; 4 Division of Bioinformatics, Medical Institute of Bioregulation, Kyushu University, Fukuoka, Japan; 5 Division of Diagnostic Pathology, Keio University School of Medicine, Shinjuku-ku, Tokyo Japan; 6 Department of Surgery, Keio University School of Medicine, Shinjuku-ku, Tokyo Japan

**Keywords:** Gastric cancer, Tumour virus infections

## Abstract

Latent Epstein-Barr Virus (EBV) infection promotes cancers derived from B-lymphocytes and epithelial cells by mechanisms that largely remain unclear. EBV-encoded nuclear antigen 1 (EBNA1) is uniformly expressed in EBV-associated cancers; however, how EBNA1 contributes to cancer progression is not known. The *MUC1* gene evolved in mammals to protect barrier tissues from viral infections. We report that *MUC1* is upregulated in EBV-associated gastric cancers (EBVaGCs). Our results demonstrate that EBNA1 and the oncogenic MUC1-C subunit form an auto-regulatory complex that controls expression of EBNA1, MUC1-C and host cellular genes. EBNA1 appropriates MUC1-C to (i) induce DNA methyltransferase (DNMT) expression and DNA methylation, (ii) suppress *CDKN1A* encoding p21 to promote proliferation, and (iii) upregulate survivin to confer survival. MUC1-C is also co-opted for localization of EBNA1 in chromatin, expression of EBV latency genes and suppression of lytic genes. Targeting MUC1-C thereby induces the switch of EBV latency to activation of the lytic phase. We further demonstrate that MUC1-C is necessary for EBVaGC stem cell (CSC) state as evidenced by regulation of NOTCH stemness genes and self-renewal capacity. These findings and the demonstration that EBV positivity has no significant effect on survival of patients with GCs indicate that EBNA1 exploits MUC1-C to maintain EBV latency and that prolonged activation of MUC1-C in response to chronic EBV infection promotes EBVaGC malignant progression.

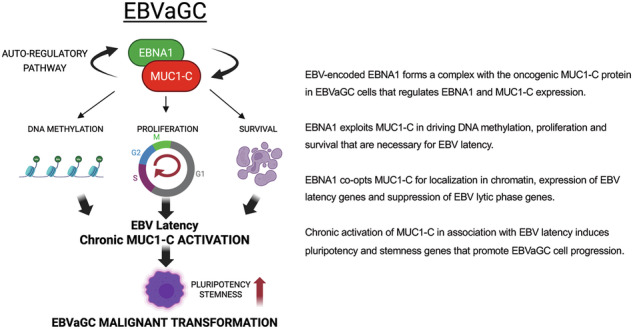

## Introduction

Epstein-Barr Virus (EBV) was discovered in 1964 in cells cultured from a Burkitt’s Lymphoma (BL) biopsy [1]. EBV infects about 95% of the adult human population throughout the world [2]. EBV infections of the B-lymphocyte pool persist asymptomatically in most humans, whereas dysregulation of the virus is a causative factor in benign diseases, such as mononucleosis, and in diverse cancers [2]. Approximately 250,000 cases of cancer and 2% of cancer deaths each year are attributed to EBV-associated malignancies [3]. As first reported for BL, EBV has been linked to Hodgkin’s lymphoma (HL), Diffuse Large B-Cell Lymphoma (DLBCL) and other T-cell and NK-cell lymphomas [2]. EBV also contributes to the pathogenesis of epithelial carcinomas that include about 10% of gastric carcinomas [4] and ~95% of undifferentiated nasopharyngeal carcinomas (NPCs) in the Far East [5]. EBV-associated gastric cancers (EBVaGCs) manifest a viral latency II program in which EBV-encoded nuclear antigen 1 (EBNA1) and, more variably, latent membrane proteins (LMP1 and 2) and small non-coding RNAs (EBERs) are expressed in the absence of other viral genes [6]. Of these, EBNA1 is the only EBV protein (i) required for viral DNA replication during latent infection, and (ii) uniformly expressed in EBV-associated cancers [7–10]. Despite decades of EBV research, it remains unclear how EBNA1 contributes to cancer progression [10, 11].

The *MUC1* gene evolved in mammals to protect barrier tissues from infections with viruses and other pathogenic organisms [12, 13]. *MUC1* encodes N-terminal (MUC1-N) and C-terminal (MUC1-C) subunits [13]. The transmembrane MUC1-C subunit is activated by disruption of homeostasis and in turn promotes inflammatory, proliferative and repair responses [13, 14]. MUC1-C regulates gene expression by driving epigenetic reprogramming and chromatin remodeling to reestablish homeostasis [13–17]. These genomic alterations are theoretically reversible; however, prolonged activation of MUC1-C in settings of chronic infection and inflammation promotes cancer progression [13]. Dysregulation of MUC1-C has been identified in pan-cancers originating in epithelial and hematopoietic tissues [13, 14]. These cancers are dependent on MUC1-C for self-renewal capacity and the cancer stem cell (CSC) state [18, 19]. Cancer progression is associated with localization of MUC1-C in chromatin, where it interacts with transcription factors and epigenetic effectors of gene expression [20, 21]. There is no reported involvement of MUC1-C in EBV-associated cancers.

In the present work focusing on EBVaGC [22], we demonstrate that EBNA1 forms a complex with MUC1-C that regulates EBNA1 and MUC1-C expression. In this way, EBNA1 subverts MUC1-C in regulating DNA methylation, proliferation and survival. Our results also show that EBNA1 exploits MUC1-C to promote EBV latency and suppress expression of EBV lytic phase genes. Consistent with a role in linking chronic inflammation with cancer, we show that MUC1-C, but not EBNA1, is necessary for EBVaGC cell stemness gene expression and self-renewal capacity. We further demonstrate that EBV positivity has no apparent effect on survival of GC patients. These findings indicate that EBNA1 appropriates MUC1-C to maintain EBV latency and highlight the importance of MUC1-C in linking EBV-associated chronic inflammation with progression of the EBVaGC CSC state.

## Methods

### Cell culture

Human YCCEL1 EBVaGC cells derived from a metastatic EBVaGC were obtained from Dr. P. Lieberman, Wistar Institute, Philadelphia, PA, USA. SNU-719 cells derived from a primary EBVaGC, AGS gastric cancer cells and AGS/EBV-BX cells obtained from the Gewurz laboratory were cultured in RPMI1640 medium (Corning Life Sciences, Corning, NY, USA) containing 10% heat-inactivated fetal bovine serum (FBS; GEMINI Bio-Products, West Sacramento, CA, USA). Authentication of the cells was performed by short tandem repeat (STR) analysis. Cells were monitored for mycoplasma contamination using the MycoAlert Mycoplasma Detection Kit (Lonza, Rockland, MA, USA). Cells were maintained for 3 months when performing experiments.

### Gene silencing and rescue

MUC1shRNA (MISSION shRNA TRCN0000122938; Sigma, St. Louis, MO, USA) or a control scrambled shRNA (CshRNA; Sigma) was inserted into the pLKO-tet-puro vector (Plasmid #21915; Addgene, Cambridge, MA, USA) as described [20]. The MUC1shRNA#2 (MISSION shRNA TRCN0000430218) was produced in HEK293T cells as described [20]. Flag-tagged MUC1-CD was inserted into pInducer20 (Plasmid #44012, Addgene) as described [20]. Cells transduced with the vectors were selected for growth in 1–2 μg/ml puromycin. Cells were treated with 0.1% DMSO as the vehicle control or 500 ng/ml DOX (Millipore Sigma, Burlington, MA, USA). Amino acids 379–386 and 451–641 of EBNA1 were in-frame fused to create the EBNA1-DN vector as described [23]. The DNA fragment was then inserted into the TRC313 vector [24]. EBNA1-DN, EBNA1 (Plasmid #37954, Addgene) and EBNA1shRNA [25] vectors were transfected into cells using Lipofectamine 3000 (Invitrogen, Waltham, MA, USA).

### Quantitative reverse-transcription PCR (qRT-PCR)

Total cellular RNA was isolated using Trizol reagent (Thermo Fisher Scientific, Waltham, MA, USA). cDNAs were synthesized using the High-Capacity cDNA Reverse Transcription Kit (Applied Biosystems, Grand Island, NY, USA) as described [20]. The cDNA samples were amplified using the Power SYBR Green PCR Master Mix (Applied Biosystems) and the CFX96 Real-Time PCR System (BIO-RAD, Hercules, CA, USA) as described [20]. Primers used for qRT-PCR are listed in Supplementary Table S1. Each experiment was performed with 3-4 independent technical replicate.

### Immunoblot analysis

Total lysates prepared from non-confluent cells were analyzed by immunoblotting with anti-MUC1-C (16564, 1:1000 dilution; Cell Signaling Technology (CST), Danvers, MA, USA and HM-1630-P1ABX, 1:1000 dilution; Thermo Fisher Scientific, Waltham, MA, USA), anti-β-actin (A5441, 1:5000 dilution; Sigma-Aldrich, Burlington, MA, USA), anti-E2F1 (3742, 1:1000 dilution; CST), anti-EBNA1 (sc-81581, 1:200 dilution; Santa Cruz, Dallas, TX, USA and ab316860, 1:1000 dilution; abcam, Cambridge, MA, USA), anti-DNMT1 (5032, 1:1000 dilution; CST), anti-DNMT3A (3598, 1:1000 dilution; CST), anti-DNMT3B (67259, 1:1000 dilution; CST), anti-PLOD1 (29480-1-AP, 1:100 dilution; Proteintech, Rosemont, IL, USA), anti-E-Cadherin (3195, 1:1000 dilution; CST), anti-CTCF (3418, 1:1000 dilution; CST), anti-RAD21 (ab992, 1:1000 dilution; Abcam), anti-PARP1 (9532, 1:1000; CST), anti-p21 (2947, 1:1000 dilution; CST), anti-survivin (2808, 1:1000 dilution; CST), anti-BCL-XL (2764, 1:1000 dilution; CST), anti-SOX2 (3579, 1:1000 dilution; CST), anti-KLF4 (12173, 1:1000 dilution; CST), anti-MYC (5605, 1:1000 dilution; CST), anti-BZLF1 (sc-53904, 1:500 dilution; Santa Cruz), anti-BMRF1 (sc-58121, 1:500 dilution; Santa Cruz), anti-NOTCH1 (3608, 1:1000 dilution; CST), anti-NOTCH2 (5732, 1:1000 dilution; CST), anti-NOTCH3 (5276, 1:1000 dilution; CST), anti-HEY1 (19929-1-AP, 1:2,000 dilution; Proteintech), anti-α-Tubulin (2144, 1:1000 dilution; CST), anti-VDAC (ab15895; 1:1000 dilution; Abcam), Lamin B1 (66095-1-Ig, 1:1000 dilution; Proteintech) and anti-Histone H3 (ab1791, 1:5000 dilution; Abcam).

### Flow cytometry

Cells were harvested with 0.05% TrypLE (Gibco, Waltham, MA, USA), washed with PBS. Cells were stained with FxCycle™ PI/RNase Staining Solution (cat. #F10797; Invitrogen, Waltham, MA, USA) for 30 min at room temperature. Data were acquired using a MACSQuant Analyzer 10 (Miltenyi Biotec, Charlestown, MA, USA) with 20,000 events per sample and analyzed using FlowJo v10.6.2 (BD Biosciences, Woburn, MA, USA). Each experiment was performed with 3 independent biological replicates.

### RNA-seq analysis

Total RNA from cells cultured in biological triplicates was isolated using the RNeasy Plus Mini Kit (Qiagen, Hilden, Germany). TruSeq Stranded mRNA (Illumina, San Diego, CA, USA) was used for library preparation as described [20]. Raw sequencing reads were aligned to the human genome (GRCh38.74) with STAR as described [20]. Gene counts were normalized and differential expression analysis was performed using DESeq2 as described [20]. The fgsea (v1.8.0) package in R was used for differential expression rank order and GSEA. Gene sets queried included those available through the Molecular Signatures Database (MsigDB) as described [20]. The Asian Cancer Research Group (ACRG) robust multi-array analysis (RMA) normalized microarray expression data for EBV-negative (*n* = 216) and EBV-positive (*n* = 16) primary gastric adenocarcinomas were retrieved from the Gene Expression Omnibus (GSE62254) using the GEOquery Bioconductor R package. Differential gene expression analysis of microarray data was performed with the limma Bioconductor R package. TCGA gene expression data and associated clinical data for GC primary tumors (*n* = 375) and normal gastric tissue (*n* = 35) were retrieved from the National Cancer Institute (NCI) Genomic Data Commons (GDC) using the TCGAbiolinks Bioconductor R package. Specifically, the harmonized data for gene expression quantification from the “STAR-Counts” standardized workflow were collected and merged with EBV viral load data as described [26].

### Coimmunoprecipitation studies

Protein lysates were immunoprecipitated with anti-MUC1-C (HM-1630-P1ABX; Thermo Fisher Scientific, Waltham, MA, USA) or a rabbit isotype control IgG (3900S, Cell Signaling Technology (CST), Danvers, MA, USA) using the Pierce Classic Magnetic Co-IP Kit (Thermo Fisher Scientific).

### DNA methylation analysis

Cells were treated with (i) vehicle or DOX for 7 days, and (ii) 0.5 μM decitabine (DAC; Sigma, St. Louis, MO) as a positive control. Genomic DNA was extracted from cells using the DNeasy Blood and Tissue kit (Qiagen, Hilden, Germany). Serially diluted genomic DNA was spotted onto a Hybond-N+ membrane (PerkinElmer, MA, USA). Membranes were incubated with anti-5-methylcytosine (5-mC) monoclonal antibody (ab10805, 1:1000 dilution; Abcam, Cambridge, MA, USA). Dot blots were developed by incubation with ECL chemiluminescence for 1 min. Images were captured and analyzed by Li-Cor Fc platform.

### Quantification of EBV genome copy number

Total DNA from 0.5–1 × 10^6^ cells extracted with the DNeasy Blood & Tissue Kit (Qiagen, Hilden, Germany) was diluted to 10 ng/ml before qPCR analysis with a primer pair targeting the EBV BALF5 gene or host β-actin promoter (Supplemental Table S2) as described [27]. Quantitative real-time PCR was performed using the Power SYBR Green PCR Master Mix (Applied Biosystems, Grand Island, NY, USA) on a CFX96 Touch Real-Time PCR Detection System (BioRad, Hercules, CA, USA). Viral DNA copy number per 1 μg DNA was calculated by interpolation of Cq values to a standard curve generated by serial dilutions of 25 ng/ml pHAGE-BALF5 plasmid as described [27]. Each experiment was performed with 3 independent technical replicates.

### Colony formation assays

Cells (3–5 × 10^3^) were seeded in 24-well plates. After 7–14 days, cells were stained with 0.5% crystal violet (LabChem, Zelienople, PA, USA) in 25% methanol as described [20]. Each experiment was performed with three independent biological replicates.

### Tumorsphere formation assays

Cells (1–3 × 10^4^) were seeded per well in 6-well ultra-low attachment culture plates (Corning Life Sciences, Tewksbury, MA, USA) in DMEM/F12 50/50 medium (Corning Life Sciences) with 20 ng/ml EGF (Millipore Sigma, Burlington, MA, USA), 20 ng/ml bFGF (Millipore Sigma) and 1% B27 supplement (Gibco, Waltham, MA, USA) as described [16, 28]. Tumorspheres with a diameter >100 microns were counted under an inverted microscope. Each experiment was performed with 3 independent biological replicates.

### Immunohistochemistry (IHC)

Tumor tissue samples from patients with EBVaGC who underwent surgical resection were obtained from the Department of Surgery, School of Medicine, Keio University following institutional approval (Protocol #20190057). Informed consent was obtained from all patients included in the study. Specimens were subjected to IHC with an anti-MUC1-C rabbit monoclonal antibody (16564, 1:1000 dilution; Cell Signaling Technology (CST), Danvers, MA, heat-induced epitope retrieval, pH 6.0) and an anti-EBNA1 antibody (sc-81581, 1:1000 dilution; Santa Cruz, Dallas, TX, USA). Evaluation of MUC1-C and EBNA1 expression was assessed based on the proportion of positively-stained neoplastic cells to the total number of neoplastic cells, and then graded from 0 to 3, (0% = 0; 0 to <25% = 1; >25% = 2). Three investigators (HO, KF, and KY) including one pathologist (KY) independently evaluated all slides. If the independent assessments were not in agreement, the slides were reviewed together by the three investigators until they reached a consensus. The consensus judgments were adopted as the results.

### Immunofluorescence (IF) microscopy

YCCEL1 cells were fixed with 4% paraformaldehyde (Sigma, St. Louis, MO, USA) for 10 min at room temperature, permeabilized with 0.1% Triton X-100 (Sigma) for 10 min, and blocked using 3% normal goat serum (Gibco, Waltham, MA, USA). Cells were incubated overnight at 4 °C with anti-MUC1-C (16564, 1:1000 dilution; Cell Signaling Technology (CST), Danvers, MA) and anti-EBNA1 antibody (sc-81581, 1:1000 dilution; Santa Cruz, Dallas, TX, USA). After washing, Alexa Fluor-conjugated secondary antibodies (goat anti-rabbit IgG Alexa Fluor 488 and anti-hamster IgG Alexa Fluor 568; Abcam, Cambridge, MA, USA) were applied for 1 h at room temperature. Nuclei were counterstained with ProLong™ Diamond Antifade Mountant with DAPI (Invitrogen, Waltham, MA, USA). Images were acquired using a Zeiss LSM 980 confocal microscope. Scale bars were added using ImageJ.

### Quantification and statistical analysis

Each experiment was performed at least three times. Unpaired two-tailed *t*-tests were used to assess differences between the mean ± SD of two groups. One-way ANOVA was performed for multiple group comparisons. *P*-values were considered significant at *p* < 0.05. GraphPad Prism9 was used for all statistical analyses. Asterisks represent *P ≤ 0.05, **P ≤ 0.01, ***P ≤ 0.001, ****P ≤ 0.0001 with CI = 95%.

## Results

### MUC1-C is upregulated in EBVaGC tumors and regulates the transcriptomes of EBVaGC cells

**Fig. 1:**
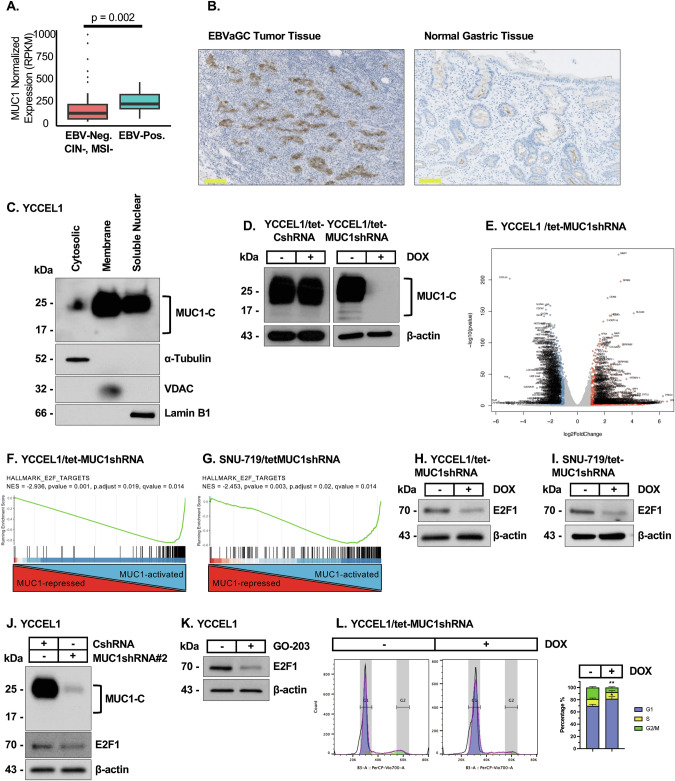
MUC1-C is upregulated in EBVaGC tumor tissues and regulates EBVaGC cell transcriptomes. **A** Analysis of the TCGA STAD dataset for MUC1 expression in EBV-positive vs EBV-negative, CIN-negative and MSI-negative GCs**. B** IHC detection of MUC1-C expression in metastatic EBVaGC tumor tissue and normal gastric epithelium. The bar represents 100 μm. **C** YCCEL1 cytosolic, membrane and soluble nuclear fractions were immunoblotted with antibodies against the indicated proteins. **D** Lysates from YCCEL1/tet-CshRNA and YCCEL1/tet-MUC1shRNA cells treated with vehicle or DOX for 7 days were immunoblotted with antibodies against the indicated proteins. **E** Volcano plot of down- and up-regulated genes in YCCEL1/tet-MUC1shRNA cells treated with vehicle of DOX for 7 days. GSEA of RNA-seq data from YCCEL1 (**F**) and SNU-719 (**G**) cells with MUC1-C silencing using the using the HALLMARK E2F TARGETS gene signature. Lysates from YCCEL1/tet-MUC1shRNA (**H**) and SNU-719/tet-MUC1shRNA (**I**) cells treated with vehicle of DOX for 7 days were immunoblotted with antibodies against the indicated proteins. **J** Lysates from YCCEL1/CshRNA and YCCEL1/MUC1shRNA#2 cells were immunoblotted with antibodies against the indicated proteins. **K** Lysates from YCCEL1 cells treated with 5 μM GO-203 for 4 days were immunoblotted with antibodies against the indicated proteins. **L** YCCEL1/tet-MUC1shRNA cells were treated with vehicle or DOX for 7 days and analyzed for cell cycle distribution by flow cytometry. The results (mean ± SD of three determinations) are expressed as the percentage of cells in G1, S and G2/M phases.

Previous work in cell-based models demonstrated that the EBV LMP1 protein induces MUC1 expression and the EMT phenotype [29]. However, it is not known if MUC1 is upregulated in EBVaGCs. Here, analysis of the TCGA gastric adenocarcinoma dataset demonstrated that MUC1 expression is significantly increased in (i) GCs vs normal gastric tissues (Supplementary Fig. S1A) and (ii) EBVaGCs vs EBV-negative GCs without chromosomal instability (CIN) and microsatellite instability (MSI) (Fig. 1A).

MUC1 is translated as a single polypeptide that undergoes auto-cleavage in the endoplasmic reticulum into MUC1 N-terminal (MUC1-N) and C-terminal (MUC1-C) subunits, which in turn form a stable non-covalent complex at the cell membrane (Supplementary Fig. S1B) [13]. With loss of homeostasis, MUC1-N is shed from the cell surface and activated MUC1-C is transported to the nucleus (Supplementary Fig. S1B). MUC1-C is expressed as ~25 kDa glycosylated and 17 kDa unglycosylated proteins (Supplementary Fig. S1B). IHC of 15 EBVaGC tissues further detected upregulation of MUC1-C expression in cell membranes and cytoplasm in 14 (93%) of the tumor samples compared to that in normal gastric epithelium (Fig. 1B; Supplementary Fig. S1C). In YCCEL1 EBVaGC cells, MUC1-C was detectable in the cell membrane, cytosolic and nuclear fractions (Fig. 1C).

To assess the functional significance of MUC1-C expression, we established YCCEL1 cells expressing tet-CshRNA or tet-MUC1shRNA. Treatment with DOX selectively downregulated MUC1-C expression in YCCEL1/tet-MUC1shRNA cells (Fig. 1D). RNA-seq performed on DOX-treated YCCEL1/tet-MUC1shRNA cells identified 643 downregulated and 599 upregulated genes with MUC1-C silencing (Fig. 1E). As a second model, we studied SNU-719 EBVaGC cells, which also express MUC1-C, although at lower levels than in YCCEL1 cells (Supplementary Fig. S1D). In SNU-719 cells with MUC1-C silencing (Supplementary Fig. S1E), 159 and 152 genes were downregulated and upregulated, respectively (Supplementary Fig. S1F). Analysis of the RNA-seq data from both cell lines with MUC1-C silencing identified (i) upregulation of the HALLMARK TNFA SIGNALING VIA NF-κB and HALLMARK EPITHELIAL MESENCHYMAL TRANSITION, and (ii) downregulation of the HALLMARK E2F TARGETS and HALLMARK G2M CHECKPOINT gene signatures (Supplementary Fig. S1G, H).

GSEA uncovered that MUC1-C drives multiple E2F target genes (Fig. 1F, G). Accordingly, we focused on E2F1, which binds directly to MUC1-C, is overexpressed in cancer, drives cell proliferation and has been linked to promoting EBV latency [30, 31]. Silencing MUC1-C in YCCEL1 and SNU-719 cells suppressed E2F1 expression (Fig. 1H, I), which was extended using an additional MUC1shRNA#2 (Fig. 1J) and the GO-203 inhibitor that targets the MUC1-C cytoplasmic domain and MUC1-C function [13] (Fig. 1K). Consistent with these results, we found that YCCEL1 and SNU-719 cells are dependent on MUC1-C for cell cycle progression (Fig. 1L; Supplementary Fig. S1I).

These findings indicate that MUC1-C (i) is upregulated in EBVaGC tumor tissues, (ii) regulates EBVaGC cell transcriptomes, and (iii) drives E2F1 expression and cell proliferation.

### MUC1-C regulates EBNA1 expression in EBVaGC cells

The EBV LMP1 protein contributes to upregulation of MUC1 expression in (i) KH-1/2 cells derived from fusions of KR-4 lymphoblastoid and HeLa cells, and (ii) EBV-infected breast cancer cells [29]. YCCEL1 cells express EBNA1 and latent membrane protein 2 (LMP2A), but not EBNA2, LMP2B or LMP1 [32]. Additionally, SNU-719 cells express EBNA1 and LMP2A, but not EBNA2 and LMP1 [33, 34]. We therefore focused here on EBNA1 based on these findings and the universal expression of this protein in EBV-associated cancers [7, 8].

**Fig. 2:**
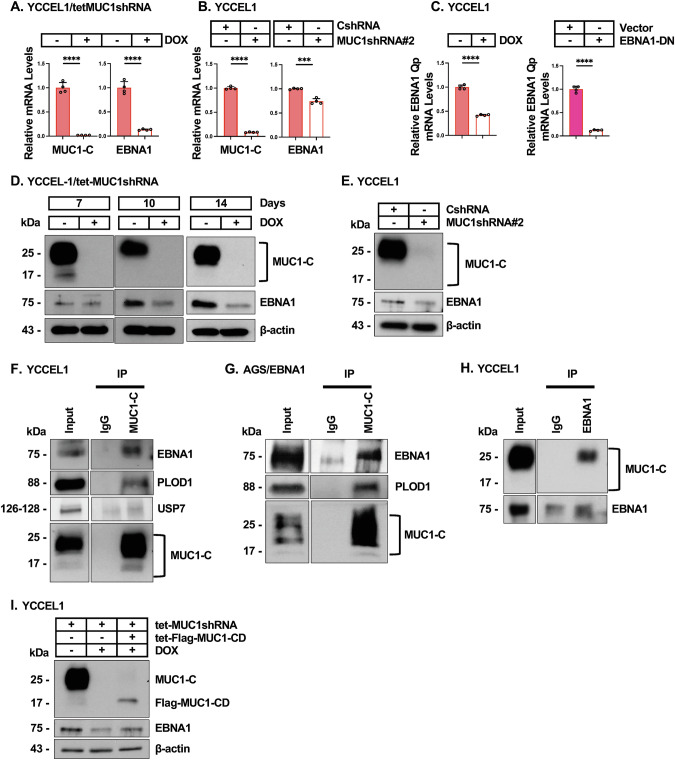
EBNA1 associates with MUC1-C in an auto-inductive pathway regulating EBNA1 and MUC1-C expression. **A** YCCEL1/tet-MUC1shRNA cells treated with vehicle or DOX for 14 days were analyzed for MUC1-C and EBNA1 transcripts by qRT-PCR. The results (mean±SD of four determinations) are expressed as relative levels compared to that obtained for vehicle-treated cells (assigned a value of 1). **B** YCCEL1/CshRNA and YCCEL1/MUC1shRNA#2 cells were analyzed for MUC1-C and EBNA1 transcripts by qRT-PCR. The results (mean±SD of four determinations) are expressed as relative levels compared to that obtained for CshRNA cells (assigned a value of 1). **C** YCCEL1/tet-MUC1shRNA cells treated with vehicle or DOX for 7 days were analyzed for EBNA1 Qp transcripts (left). YCCEL1/empty vector and YCCEL1/EBNA1-DN cells were analyzed for EBNA1 Qp transcripts (right). The results (mean±SD of four determinations) are expressed as relative EBNA1 Qp mRNA levels compared to that obtained for vehicle-treated/empty vector cells (assigned a value of 1). **D** Lysates from YCCEL1/tet-MUC1shRNA cells treated with vehicle or DOX for 7, 10 and 14 days were immunoblotted with antibodies against the indicated proteins. **E** Lysates from YCCEL1/CshRNA and YCCEL1/MUC1shRNA#2 were immunoblotted with antibodies against the indicated proteins. **F** Lysates from YCCEL1 cells were precipitated with anti-MUC1-C or a control IgG. The precipitates and input lysate were immunoblotted with antibodies against the indicated proteins. **G** Lysates from AGS/EBNA1 cells were precipitated with anti-MUC1-C or a control IgG. The precipitates and input lysate were immunoblotted with antibodies against the indicated proteins. **H** Lysates from YCCEL1 cells were precipitated with anti-EBNA1 or a control IgG. The precipitates and input lysate were immunoblotted with antibodies against the indicated proteins. **I** Lysates from YCCEL1 cells expressing the designated vectors were treated with vehicle or DOX for 14 days and immunoblotted with antibodies against the indicated proteins.

Unexpectedly, we found that DOX-inducible MUC1-C silencing in YCCEL1 (Fig. 2A) and SNU-719 (Supplementary Fig. S2A) cells decreases EBNA1 transcripts. Targeting MUC1-C with a second MUC1shRNA (Fig. 2B) confirmed that MUC1-C drives EBNA1 mRNA levels. EBNA1 suppresses transcription of the *EBNA1* gene by binding to the Q promoter (Qp) in an auto-regulatory pathway [10]. This suppression of Qp is overcome by E2F-mediated displacement of EBNA1 and in turn Qp activation [10]. Analysis of Qp-initiated EBNA1 transcripts demonstrated that silencing MUC1-C suppresses Qp activity (Fig. 2C, left). As a control, similar results were obtained with an EBNA1 dominant negative (EBNA1-DN)(Fig. 2C, right). In concert with the demonstration that MUC1-C induces Qp activation and EBNA1 mRNA levels, we found that silencing MUC1-C in YCCEL1 cells for 7–14 days decreases EBNA1 protein levels (Fig. 2D, E).

Coimmunoprecipitation studies further demonstrated that MUC1-C forms complexes with EBNA1 in YCCEL1 and SNU-719 cells (Fig. 2F; Supplementary Fig. S2B). EBNA1 is stabilized by the PLOD1 lysine hydroxylase [35] and the ubiquitin-specific protease 7 (USP7) [36]. PLOD1, but not USP7, was detectable in the anti-MUC1-C precipitates (Fig. 2F; Supplementary Fig. S2B). We also found that silencing MUC1-C suppresses PLOD1 transcripts (Supplementary Fig. S2C) and protein levels (Supplementary Figs. S2D, E). By contrast, silencing MUC1-C had no apparent effect on USP7 expression (Supplementary Figs. S2D, E), indicating that MUC1-C selectively regulates the EBNA1/PLOD1 pathway. Studies of EBV-negative AGS cells transfected to express EBNA1 confirmed that MUC1-C forms complexes with EBNA1 and PLOD1 (Fig. 2G). In the reciprocal experiment, analysis of anti-EBNA1 immunoprecipitates demonstrated that EBNA1 associates with MUC1-C (Fig. 2H).

MUC1-C consists of a 72 aa intrinsically disordered cytoplasmic domain (CD) (Supplementary Fig. S1B) [13]. Expression of Flag-tagged MUC1-C/CD revealed that the MUC1-C cytoplasmic domain is sufficient for forming a complex with EBNA1 (Supplementary Fig. S2F). Studies were also performed with GST-MUC1-CD and purified EBNA1 using an approach previously described for detecting direct binding to MYC [37]. In contrast to MYC, there was no detectable binding of MUC1-CD and EBNA1, indicating that the association of MUC1-C and EBNA1 in cells is indirect. Nonetheless, rescuing MUC1-C silencing with MUC1-CD recovered EBNA1 expression (Fig. 2I). These findings that MUC1-C (i) induces Qp activation and EBNA1 transcripts, (ii) associates with EBNA1, and (iii) increases PLOD1 levels, support a model in which MUC1-C regulates EBNA1 expression by transcriptional and post-translational mechanisms.

### EBNA1 exploits MUC1-C by forming an auto-regulatory pathway that controls EBNA1 and host cellular gene expression

**Fig. 3:**
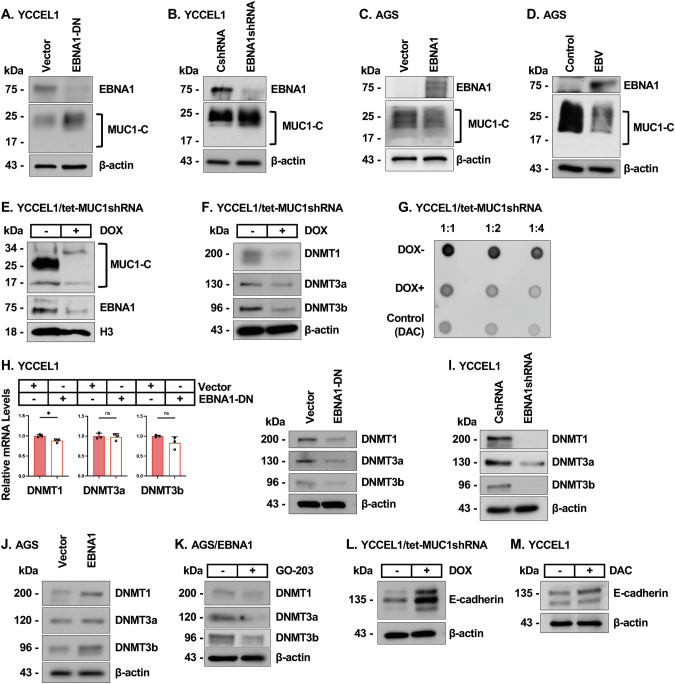
EBNA1 regulates MUC1-C and DNMT expression. **A** Lysates from YCCEL1 cells expressing an empty vector or EBNA1-DN were immunoblotted with antibodies against the indicated proteins. **B** Lysates from YCCEL1/CshRNA and YCCEL1/EBNA1shRNA cells were immunoblotted with antibodies against the indicated proteins. **C** Lysates from AGS/vector and AGS/EBNA1 cells were immunoblotted with antibodies against the indicated proteins. **D** Lysates from AGS and AGS/EBV cells were immunoblotted with antibodies against the indicated proteins. **E** Chromatin from YCCEL1/tet-MUC1shRNA cells treated with vehicle or DOX for 7 days was immunoblotted with antibodies against the indicated proteins. **F** Lysates from YCCEL1/tet-MUC1shRNA cells treated with vehicle or DOX for 7 days were immunoblotted with antibodies against the indicated proteins. **G** YCCEL1/tet-MUC1shRNA cells treated with vehicle of DOX for 7 days were analyzed for global DNA methylation. YCCEL1 cells treated with decitabine (DAC) were analyzed as a control. **H** YCCEL1/vector and YCCEL1/EBNA1-DN cells were analyzed for DNMT1, DNMT3a and DNMT3b transcripts by qRT-PCR (left). The results (mean±SD of three determinations) are expressed as relative levels compared to that obtained for vehicle-treated cells (assigned a value of 1). Lysates were immunoblotted with antibodies against the indicated proteins (right). **I** Lysates from YCCEL1/CshRNA and YCCEL1/EBNA1shRNA cells were immunoblotted with antibodies against the indicated proteins. **J** Lysates from AGS/vector and AGS/EBNA1 cells were immunoblotted with antibodies against the indicated proteins. **K** Lysates from AGS/EBNA1 cells treated with 3 μM GO-203 for 4 days were immunoblotted with antibodies against the indicated proteins. **L**. Lysates from YCCEL1/tet-MUC1shRNA cells treated with vehicle or DOX for 7 days were immunoblotted with antibodies against the indicated proteins. **M** Lysates from YCCEL1 cells treated with vehicle or 0.5 μM DAC for 3 days were immunoblotted with antibodies against the indicated proteins.

In investigating if EBNA1 regulates MUC1-C, we found that expression of a EBNA1-DN vector is associated with increases in MUC1-C levels (Fig. 3A; Supplementary Fig. S3A). Silencing EBNA1 with an EBNA1shRNA further demonstrated upregulation of MUC1-C expression (Fig. 3B). Moreover, studies of AGS/EBNA1 cells (Fig. 3C) and AGS cells infected with EBV (AGS/EBV) confirmed suppression of MUC1-C levels (Fig. 3D). These results and those demonstrating that MUC1-C increases EBNA1 expression support an auto-regulatory pathway in which EBNA1 potentially co-opts MUC1-C to maintain EBNA1 at levels necessary for maintaining EBV latency, while on the other hand, restricting its expression for preventing detection by the host immune system [10].

EBNA1 sustains EBV latency by tethering the EBV genome to cellular chromatin [10, 38, 39]. Analysis of YCCEL1 cell chromatin demonstrated expression of the MUC1-C~25 kDa glycoprotein and the 17 kDa protein as monomers and 34 kDa homodimers (Supplementary Fig. S3B). In SNU-719 cell chromatin, MUC1-C was detectable as the 17 kDa monomer and 34 kDa homodimer (Supplementary Fig. S3B). We also detected EBNA1 in YCCEL1 and SNU-719 cell chromatin (Supplementary Fig. S3B), which was confirmed using 3 different approaches for purifying chromatin fractions (Supplementary Fig. S3C). Silencing MUC1-C in YCCEL1 and SNU-719 cells downregulated EBNA1 in chromatin (Fig. 3E; Supplementary Figs. S3D, E), indicating that EBNA1 could be co-opting MUC1-C in chromatin for the regulation of host gene expression.

DNA hypermethylation in EBVaGC is a well-established characteristic that has been linked to upregulation of DNA methyltransferases (DNMTs) by mechanisms that have largely remained unclear [7, 40–43]. Our prior work demonstrated that MUC1-C induces DNMT expression by an NF-κB-mediated transcriptional mechanism [15, 44, 45]. As a result, MUC1-C regulates global, as well as gene-specific DNA methylation patterns, as evidenced by analysis of LINE-1 repeat elements and promoters of the *CDH1, PTEN and BRCA1* tumor suppressor genes [15, 44, 45]. Targeting MUC1-C in YCCEL1 and SNU-719 cells downregulated DNMT1, DNMT3a and DNMT3b expression (Fig. 3F; Supplementary Fig. S3F and S3G). Consistent with these results and as previously reported in other types of cancers [15, 44, 45], silencing MUC1-C in EBVaGC cells decreased global DNA methylation levels (Fig. 3G). We also found that targeting EBNA1 has no apparent effect on DNMT1, DNMT3a and DNMT3b transcripts (Fig. 3H), but downregulates their protein levels (Fig. 3H, I; Supplementary Fig. S3H), indicating that EBNA1 regulates DNMT expression by post-transcriptional mechanisms. The effects of MUC1-C and EBNA1 on DNMT expression could conceivably be conferred by mutually exclusive pathways. To address this possibility, we studied AGS/EBNA1 cells and found that EBNA1-mediated upregulation of DNMTs (Fig. 3J) is MUC1-C-dependent as evidenced by suppression with GO-203 treatment (Fig. 3K). These results indicated that EBNA1 exploits MUC1-C in regulating DNMT expression and DNA hypermethylation.

EBNA1-driven DNA hypermethylation represses host cellular tumor suppressor genes (TSGs) in promoting latency [41, 42, 46, 47]. As one example of a TSG repressed by DNA hypermethylation in EBVaGCs, we selected *CDH1* encoding the E-cadherin effector of EMT and cancer progression [41, 42]. E-cadherin was induced by targeting MUC1-C in YCCEL1 and SNU-719 cells (Fig. 3L; Supplementary Fig. S3I). As confirmation of repression by a DNMT-mediated mechanism, treatment with the DNMT inhibitor decitabine (DAC) induced E-cadherin expression (Fig. 3M). As another example, *RHOB* is a TSG repressed by EBNA1-mediated hypermethylation that suppresses EBVaGC progression [48]. Silencing MUC1-C in YCCEL1 and SNU-719 cells derepressed RHOB expression (Supplementary Fig. S3J, K), which was confirmed to be DNMT-mediated by DAC treatment (Supplementary Fig. S3L).

These results indicate that EBNA1 diverts MUC1-C for inducing DNMT expression, DNA hypermethylation and repression of host cell tumor suppressor genes.

### EBNA1 co-opts MUC1-C for regulation of EBVaGC cell proliferation and survival genes

**Fig. 4:**
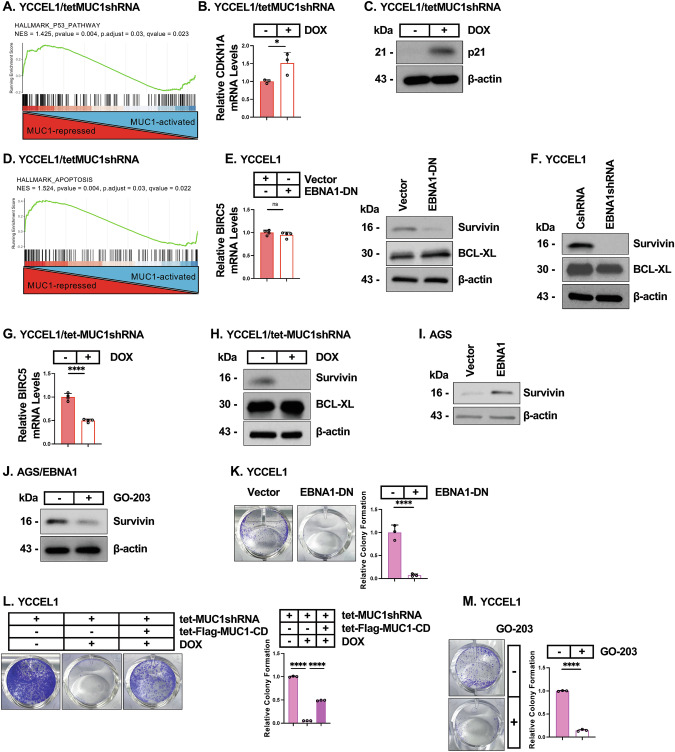
EBNA1 co-opts MUC1-C in regulating p21 and survivin expression. **A** GSEA of RNA-seq data from YCCEL1 cells with MUC1-C silencing was performed using the HALLMARK P53 PATHWAY gene signature. **B**, **C** YCCEL1/tet-MUC1shRNA cells treated with vehicle or DOX for 7 days were analyzed for CDKN1A transcripts by qRT-PCR. The results (mean±SD of three determinations) are expressed as relative levels compared to that obtained for vehicle-treated cells (assigned a value of 1) (**B**). Lysates were immunoblotted with antibodies against the indicated proteins (**C**). **D** GSEA of RNA-seq data from YCCEL1 cells with MUC1-C silencing was performed using the HALLMARK APOPTOSIS gene signature. **E** YCCEL1/vector and YCCEL1/EBNA1-DN cells were analyzed for BIRC5 transcripts by qRT-PCR (left). The results (mean±SD of four determinations) are expressed as relative levels compared to that obtained for vehicle-treated cells (assigned a value of 1). Lysates were immunoblotted with antibodies against the indicated proteins (right). **F** Lysates from YCCEL1/CshRNA and YCCEL1/EBNA1shRNA cells were immunoblotted with antibodies against the indicated proteins. **G**, **H** YCCEL1/tet-MUC1shRNA cells treated with vehicle or DOX for 7 days were analyzed for BIRC5/survivin transcripts by qRT-PCR. The results (mean±SD of four determinations) are expressed as relative levels compared to that obtained for vehicle-treated cells (assigned a value of 1) (**G**). Lysates were immunoblotted with antibodies against the indicated proteins (**H**). **I** Lysates from AGS/vector and AGS/EBNA1 cells were immunoblotted with antibodies against the indicated proteins. **J** Lysates from AGS/EBNA1 cells treated with vehicle or 3 μM GO-203 for 4 days were immunoblotted with antibodies against the indicated proteins. **K** YCCEL1/vector and YCCEL1/EBNA1-DN cells were analyzed for colony formation. Shown are representative photomicrographs of stained colonies (left). The results (mean±SD of three determinations) are expressed as relative colony formation compared to that for vector cells (assigned a value of 1)(right). **L** YCCEL1 cells expressing the designated vectors were treated with vehicle or DOX for 7 days and analyzed for colony formation. Shown are representative photomicrographs of stained colonies (left). The results (mean±SD of three determinations) are expressed as relative colony formation compared to that for control cells (assigned a value of 1)(right). **M** YCCEL1 cells treated with vehicle or 5 μM GO-203 for 7 days were analyzed for colony formation. Shown are representative photomicrographs of stained colonies (left). The results (mean ± SD of three determinations) are expressed as relative colony formation compared to that for vector cells (assigned a value of 1) (right).

Having found that EBNA1 exploits MUC1-C for the regulation of host cell genes, we next turned to the p53 pathway. EBNA1 suppresses the p53 pathway to maintain EBV latency by promoting host cell proliferation and survival [8, 10]. We found that MUC1-C is associated with suppression of the HALLMARK P53 PATHWAY gene signature in YCCEl1 and SNU-719 cells (Fig. 4A; Supplementary Fig. S4A). Among shared upregulated p53-target genes in YCCEL1 and SNU-719 cells, silencing MUC1-C resulted in induction of *CDKN1A* encoding the p21 cyclin-dependent kinase inhibitor (Fig. 4B, C; Supplementary Fig. S4B, C), which like p53 regulates host cell proliferation. We also found that EBNA1 suppresses p21 protein, but not mRNA levels, in support of a post-transcriptional mechanism (Supplementary Fig. S4D).

E2F and p53 cooperatively restrict tumorigenesis by integrating proliferation with cell survival [30, 49]. Given that MUC1-C regulates the E2F and p53 pathways, we reasoned that EBNA1 could be co-opting MUC1-C as a means of controlling this restriction point. Along this line of thinking, we found that silencing MUC1-C in YCCEL1 and SNU-719 cells associates with regulation of the HALLMARK APOPTOSIS gene signature (Fig. 4D; Supplementary Fig. S4E). We therefore focused on *BIRC5* based on the finding that EBNA1 confers resistance to apoptosis by upregulating survivin in B-lymphoma cells [50]. Suppressing EBNA1 in YCCEL1 cells decreased survivin levels, but not that of the BCL-XL anti-apoptotic protein as a control (Fig. 4E, F). Targeting MUC1-C genetically and pharmacologically in YCCEL1 and SNU-719 cells similarly decreased survivin expression (Fig. 4G, H; Supplementary Figs. S4F–I). In addressing whether EBNA1 exploits MUC1-C to regulate survivin, we found that EBNA1-mediated induction of survivin (Fig. 4I) is suppressed by targeting MUC1-C function (Fig. 4J). These results collectively supported a pathway in which EBNA1 upregulates survivin by a MUC1-C-dependent mechanism.

We therefore assessed dependence on EBNA1 and MUC1-C for survival and found that EBNA1-DN expression suppresses colony formation of YCCEL1 and SNU-719 cells (Fig. 4K; Supplementary Fig. S4J). Silencing MUC1-C also inhibited clonogenic survival, which was confirmed to be MUC1-C-dependent as evidenced by rescue with MUC1-CD (Fig. 4L; Supplementary Fig. S4K). Furthermore, treatment with GO-203 inhibited clonogenicity of YCCEL1 and SNU-719 cells (Fig. 4M; Supplementary Fig. S4L).

These findings indicate that EBNA1 exploits MUC1-C for (i) suppression of p53 signaling and p21 expression in promoting proliferation, and (ii) induction of survivin in conferring survival.

### MUC1-C maintains the EBV latency phase in EBVaGC cells

**Fig. 5:**
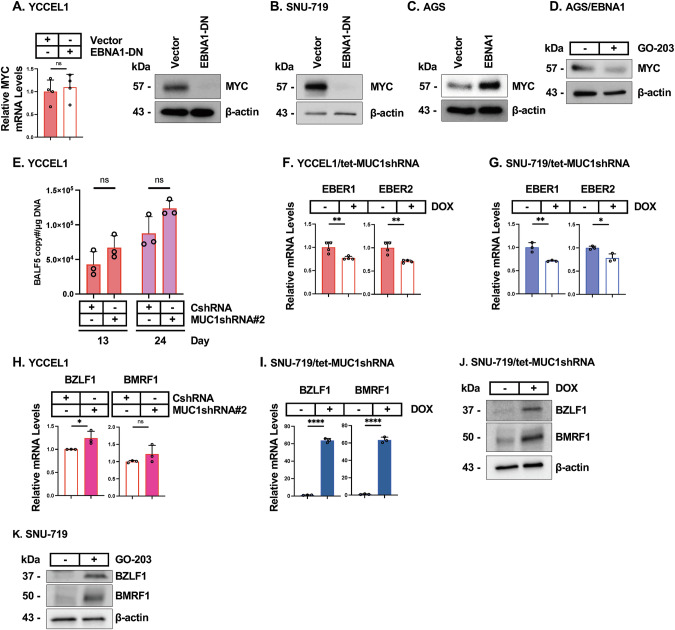
MUC1-C regulates EBNA1 expression in chromatin and EBV latency in EBVaGC cells. **A** YCCEL1/vector and YCCEL1/EBNA1-DN cells were analyzed for MYC transcripts by qRT-PCR (left). The results (mean±SD of four determinations) are expressed as relative levels compared to that obtained for vehicle-treated cells (assigned a value of 1). Lysates from YCCEL1/vector and YCCEL1/EBNA1-DN cells were immunoblotted with antibodies against the indicated proteins (right). **B** Lysates from SNU-719/vector and SNU-719/EBNA1-DN cells cells were immunoblotted with antibodies against the indicated proteins. **C** Lysates from AGS/vector and AGS/EBNA1 cells were immunoblotted with antibodies against the indicated proteins. **D** Lysates from AGS/EBNA1 cells treated with vehicle or 3 μM GO-203 for 4 days were immunoblotted with antibodies against the indicated proteins. **E** YCCEL1/CshRNA and YCCEL1/MUC1shRNA#2 cells were analyzed for EBV copy number on days 13 and 24. The results (mean±SD of three determinations) are expressed as BALF5 copy number/μg DNA. YCCEL1/tet-MUC1shRNA (**F**) and SNU-719/tet-MUC1shRNA (**G**) cells treated with vehicle or DOX for 7 days were analyzed for the indicated transcripts by qRT-PCR. The results (mean±SD of three or four determinations) are expressed as relative levels compared to that obtained for vehicle-treated cells (assigned a value of 1). **H** YCCEL1/CshRNA and YCCEL1/MUC1shRNA#2 cells were analyzed for the indicated transcripts by qRT-PCR. The results (mean±SD of three determinations) are expressed as relative levels compared to that obtained for vehicle-treated cells (assigned a value of 1). SNU-719/tet-MUC1shRNA cells treated with vehicle or DOX for 7 days were analyzed for the indicated transcripts by qRT-PCR. The results (mean±SD of three determinations) are expressed as relative levels compared to that obtained for vehicle-treated cells (assigned a value of 1) (**I**). Lysates were immunoblotted with antibodies against the indicated proteins (**J**). **K** Lysates from SNU-719 cells treated with vehicle or 3 μM GO-203 for 2 days were immunoblotted with antibodies against the indicated proteins.

The mechanisms that maintain EBV latency are complex [27, 51–53]. EBNA1 integrates hypermethylation of the EBV genome with regulation of viral latency genes [7–10, 54]. MYC has also been linked to regulating EBV latency in B cells by binding to the viral genome [27]. MUC1-C drives *MYC* expression by activation of the beta-catenin/TCF4 pathway [13]. Here, we found that targeting EBNA1 in YCCEL1 and SNU-719 cells markedly downregulates MYC protein levels (Fig. 5A, B). Consistent with these results, AGS/EBNA1 cells exhibited upregulation of MYC (Fig. 5C), which we found is MUC1-C-dependent (Fig. 5D). MUC1-C regulates the MYC transactivation function by directly binding to the MYC HLH-LZ domain [13, 37]. In concert with transcriptional and post-translational regulation of MYC, silencing MUC1-C suppressed the HALLMARK MYC TARGETS V1 gene signature in YCCEL1 and SNU-719 cells (Supplementary Fig. S5A, B).

The 3-dimensional structure of the EBV genome is also regulated by integration of DNA methylation with activity of CCCTC-binding factor (CTCF), cohesin, and PARP1 [46, 52, 53, 55]. CTCF and the cohesin complex, comprised of SMC1, SMC3 and RAD21 subunits, are localized at discrete EBV genomic sites. In YCCEL1 and SNU-719 cells, we found that MUC1-C regulates levels of CTCF and RAD21, but not PARP1, which is directly activated by a MUC1-C-mediated mechanism [56] (Supplementary Fig. S5C, D). These results collectively indicated that, in addition to driving DNA hypermethylation, EBNA1 exploits MUC1-C to promote EBV latency by (i) appropriating the MYC signaling pathway, and (ii) inducing expression of effectors that regulate the EBV 3-dimensional structure.

EBV latent replication is conferred by EBNA1-mediated activation of oriP and by exploiting the host cell DNA replication machinery. The EBV latency phase is maintained by ensuring that a constant EBV copy number persists with cell division. We found that silencing MUC1-C has no significant effect on EBV copy number during multiple cell divisions over 13 to 24 days (Fig. 5E), indicating that MUC1-C plays alternative roles for promoting EBV latency. Along these lines, we found that MUC1-C is necessary for expression of the EBV EBER1 and EBER2 latency genes in YCCEL1 (Fig. 5F) and SNU-719 (Fig. 5G) cells. Given the findings that MUC1-C has no apparent effect on EBV copy number, these results indicated that downregulation of the EBER1/2 transcripts is conferred by MUC1-C-mediated transcriptional and/or post-transcriptional mechanisms. In addition, silencing MUC1-C in YCCEL1 cells increased expression of the BZLF1 lytic phase inducer (Fig. 5H). This response was more pronounced in SNU-719 cells with MUC1-C silencing and included induction of the downstream BMRF1 processivity factor (Fig. 5I). By extension, targeting MUC1-C genetically and pharmacologically with GO-203 resulted in upregulation of the BZLF1 and BMRF1 proteins (Fig. 5J, K), providing further support for the importance of MUC1-C in promoting EBV latency.

### MUC1-C is necessary for the EBVaGC CSC state

**Fig. 6:**
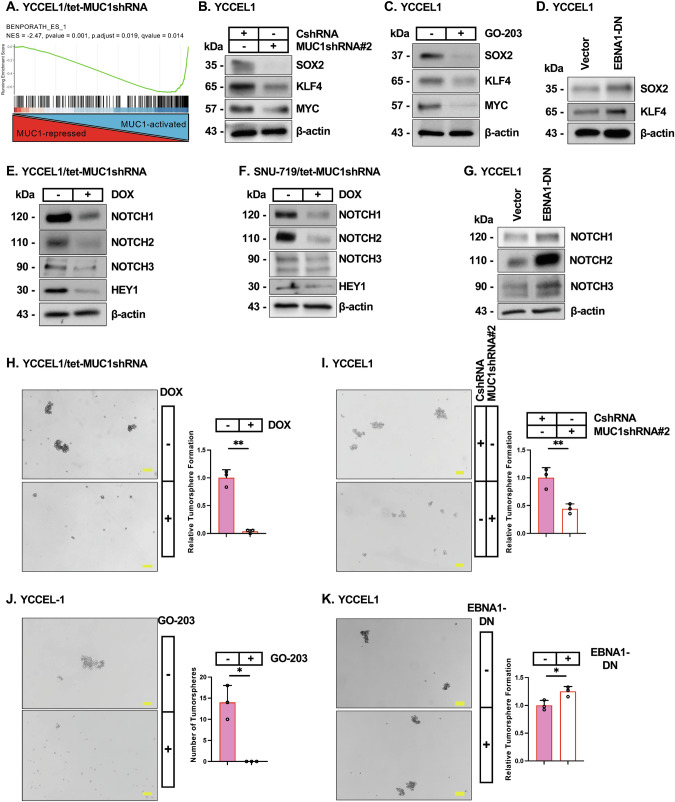
MUC1-C is necessary for the EBVaGC CSC state. **A** GSEA of RNA-seq data from YCCEL1 cells with MUC1-C silencing was performed using the BENPORATH ES1 gene signature. **B** Lysates from YCCEL1/CshRNA and YCCEL1/MUC1shRNA#2 cells were immunoblotted with antibodies against the indicated proteins. **C** Lysates from YCCEL1 cells treated with vehicle or 5 μM GO-203 for 4 days were immunoblotted with antibodies against the indicated proteins. **D** Lysates from YCCEL1/vector and YCCEL1/EBNA1-DN cells were immunoblotted with antibodies against the indicated proteins. Lysates from YCCEL1/tet-MUC1shRNA (**E**) and SNU-719/tet-MUC1shRNA (**F**) cells treated with vehicle or DOX for 7 days were immunoblotted with antibodies against the indicated proteins. **G** Lysates from YCCEL1/vector and YCCEL1/EBNA1-DN cells were immunoblotted with antibodies against the indicated proteins. **H** YCCEL1/tet-MUC1shRNA cells treated with vehicle or DOX for 7 days were analyzed for tumorsphere formation. Shown are representative photomicrographs of tumorspheres (left). The results (mean±SD of three determinations) are expressed as relative tumorsphere formation compared to that for vehicle-treated cells (assigned a value of 1)(right). **I** YCCEL1/CshRNA and YCCEL1/MUC1shRNA#2 cells were analyzed for tumorsphere formation. Shown are representative photomicrographs of tumorspheres (left). The results (mean±SD of three determinations) are expressed as relative tumorsphere formation compared to that for CshRNA cells (assigned a value of 1)(right). **J** YCCEL1 cells treated with vehicle or 5 μM GO-203 for 7 days were analyzed for tumorsphere formation. Shown are representative photomicrographs of tumorspheres (left). The results (mean±SD of three determinations) are expressed as number of tumorsphers (right). **K** YCCEL1/vector and YCCEL1/EBNA1-DN cells were analyzed for tumorsphere formation. Shown are representative photomicrographs of tumorspheres (left). The results (mean±SD of three determinations) are expressed as relative tumorsphere formation compared to that for vector cells (assigned a value of 1) (right).

EBV promotes cancer progression by maintaining the latency phase and thereby chronic inflammation [11, 57]. Prolonged activation of MUC1-C by chronic inflammation drives the CSC state [13]. Accordingly, we asked if MUC1-C contributes to EBVaGC CSC progression. GSEA of YCCEL1 and SNU-719 cell RNA-seq datasets demonstrated that MUC1-C silencing is associated with suppression of the BENPORATH ES1 gene signature (Fig. 6A; Supplementary Fig. S6A) derived from embryonic stem cells and undifferentiated cancer cells [58].

Stemness of cancer stem cells (CSCs) is associated with expression of the Yamanaka OCT4, SOX2, KLF4 and MYC (OSKM) pluripotency factors that confer lineage plasticity and dedifferentiation [59]. Targeting MUC1-C in YCCEL1 cells genetically and pharmacologically downregulated SOX2, KLF4 and MYC; whereas OCT4 was expressed constitutively at low to undetectable levels (Fig. 6B, C). Comparable results in SNU-719 cells confirmed that MUC1-C drives SOX2, KLF4 and MYC expression (Supplementary Fig. S6B). Focusing here on SOX2 and KLF4, which confer stemness in cancer [60, 61], we found that, in contrast to MUC1-C, EBNA1 suppresses their expression (Fig. 6D).

To further assess this distinction between MUC1-C and EBNA1 functions, we studied expression of *NOTCH* genes as additional effectors of the CSC state [62]. Silencing MUC1-C in YCCEL1 and SNU-719 cells decreased (i) NOTCH1-3, and (ii) the HEY1 downstream effector of the NOTCH pathway (Fig. 6E, F). By contrast, inhibiting EBNA1 increased NOTCH1-3 expression (Fig. 6G), indicating that (i) MUC1-C drives SOX2, KLF4 and NOTCH1-3 expression, and (ii) EBNA1 suppresses these effectors of stemness. In concert with these results, targeting MUC1-C genetically and pharmacologically suppressed self-renewal capacity (Fig. 6H–J), whereas, EBNA1-DN expression increased tumorsphere formation (Fig. 6K). Targeting MUC1-C in SNU-719 cells also suppressed self-renewal capacity (Supplementary Fig. S6D, E). These findings indicate that MUC1-C, but not EBNA1, is necessary for driving the CSC state in EBVaGC cells.

### Expression of genes regulated by the EBNA1/MUC1-C interaction is similar in EBV-positive and -negative GCs

**Fig. 7:**
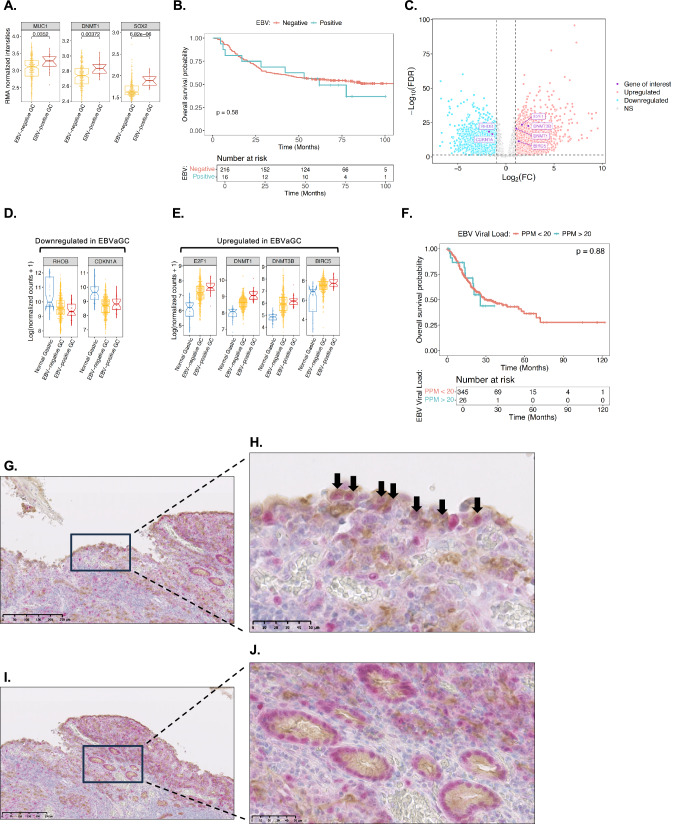
Expression of EBNA1, MUC1-C and selected co-regulated genes in EBVaGCs. **A** RMA normalized intensities of MUC1, DNMT1 and SOX2 expression from analysis of the ACRG dataset of EBV-negative and -positive GCs. **B** Overall survival of patients with EBV-negative and -positive GCs in the ACRG cohort. **C** Genes significantly downregulated (left) and upregulated (right) in EBV-positive and -negative GCs vs normal gastric tissues. Highlighted are genes identified as being regulated by EBNA1 and MUC1-C interactions. Analysis of EBV-positive GCs (n = 26), EBV-negative GCs (n = 349) and normal gastric tissue samples (n = 35) for (i) RHOB and CDKN1A (**D**), and (ii) E2F1, DNMT1, DNMT3B and BIRC5 (**E**) expression. **F** Overall survival of patients with EBV-negative and -positive GCs in the STAD cohort. Representative IHC staining of EBNA1 (red) and MUC1-C (brown) along the leading edge of an EBVaGC (**G**) with arrows highlighting co-expression in individual tumor cells (**H**). **I**, **J** Representative IHC staining of ductal structures within EBVaGCs that co-express EBNA1 (red) and MUC1-C (brown).

MUC1 expression is increased in EBV-positive and -negative GCs vs normal gastric tissues. To our knowledge, there is surprisingly no evidence that EBV positivity has an effect on the clinical outcome of patients with GCs. We therefore analyzed a dataset from the Asian Cancer Research Group (ACRG) that included complete entries for 16 EBV-positive and 216 EBV-negative gastric adenocarcinomas (GCs) (GSE62254). Robust Multi-Array Average (RMA) intensities corrected for background, normalized and log2 transformed revealed significant upregulation of MUC1 expression in EBV-positive vs EBV-negative GCs (Fig. 7A). Consistent with the demonstration that MUC1-C induces DNMT1 and SOX2 expression in EBVaGC cell lines, we also found significantly higher DNMT1 and SOX2 levels in EBV-positive GCs (Fig. 7A). Of interest, there was no significant difference in disease-free survival (DFS; Supplementary Fig. S7A) or overall survival (OS; Fig. 7B) for patients with EBV-positive and -negative GCs in this cohort.

We also analyzed the STAD dataset, which includes 26 EBV-positive GCs, 349 EBV-negative GCs and 35 normal gastric tissue samples. From this dataset, we identified genes significantly down- and up-regulated in EBV-positive and EBV-negative GCs vs normal gastric tissues (Fig. 7C). Analysis of these genes showed downregulation of RHOB and CDKN1A in both EBV-positive and -negative GCs vs normal gastric tissues (Fig. 7D). In addition, upregulation of E2F1, DNMT1, DNMT3B and BIRC5 expression was identified in both EBV-positive and -negative GCs vs normal gastric tissue (Fig. 7E). Moreover, as found from the ACRG cohort, OS of patients with EBV-positive vs EBV-negative GCs was not significantly different (Fig. 7F), indicating that EBV positivity has no apparent effect on survival of patients with GCs.

In extending these studies with IHC analysis of EBVaGCs, we found that EBNA1 is expressed within the nuclei of tumor cells, particularly those located along the leading edge (Fig. 7G). MUC1-C was also detected in these cells with diffuse staining throughout the cell membrane, cytoplasm and nucleus (Fig. 7H). By contrast, no detectable staining for EBNA1 or MUC1-C was observed in infiltrating lymphocytes (Fig. 7G, H). Similar patterns were observed with IF staining of YCCEL1 cells; that is, localization of EBNA1 to the nucleus and expression of MUC1-C in the cell membrane, cytoplasm and nucleus (Supplementary Fig. S7B). Identification of co-localized EBNA1 and MUC1-C signals was detectable to a limited extent along the nuclear membrane (Supplementary Fig. S7B), consistent with the findings that EBNA1 and MUC1-C largely interact in chromatin, which is not detectable by IF staining. EBNA1 and MUC1-C were also detectable in nests of cells within EBVaGC tumors (Fig. 7I). MUC1-C is expressed along the apical borders of ductal epithelial cells [13]. Of interest, we found co-expression of MUC1-C and EBNA1 in cells lining ductal structures within the tumors (Fig. 7J), which could represent potential settings in which EBNA1 subverts MUC1-C for driving EBV latency and EBVaGC progression.

## Discussion

**Fig. 8:**
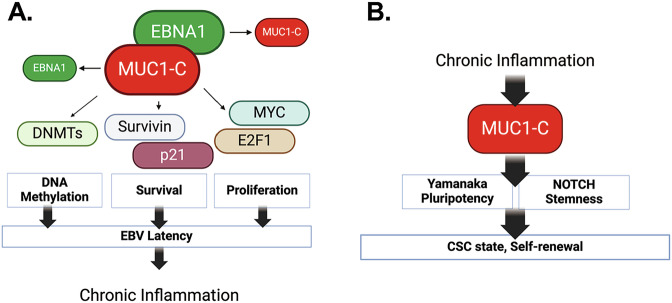
Schemas depicting EBNA1-driven appropriation of MUC1-C for promoting EBV latency and effects of associated chronic inflammation on MUC1-C activation and EBVaGC progression. **A**. EBNA1 forms a complex with MUC1-C that contributes to maintaining EBNA1 levels. In turn, EBNA1 has the capacity to fine-tune an EBNA1/MUC1-C auto-inductive pathway by regulating MUC1-C expression. EBNA1 thereby co-opts MUC1-C to drive different mechanisms that contribute to EBV latency. As one mechanism, EBNA1 exploits MUC1-C for induction of DNMT expression and global levels of DNA methylation and the repression of cellular TSGs. As a second mechanism, EBNA1 adopts MUC1-C for regulating expression of p21 and survivin in integrating proliferation and survival that are necessary for EBV latency. Of importance in addressing the impact of MUC1-C/EBNA1 interactions on gene expression, we found that targeting EBNA1 has no significant effect on (i) DNMT1, DNMT3a and DNMT3b, (ii) BIRC5/survivin, (iii) CDKN1A, and (iv) MYC transcripts, but regulates their protein levels, indicating mechanisms independent of EBNA1 transcriptional activities. These results and the demonstration that MUC1-C is necessary for the regulation of DNMT, survivin, p21 and MYC further indicate that EBNA1 is exploiting MUC1-C for their expression in promoting EBV latency and suppressing the lytic phase. **B**. Co-opting of MUC1-C to promote EBV latency has a potentially adverse outcome in driving cancer progression. Along these lines, prolonged activation of MUC1-C in response to chronic inflammation promotes the CSC state as evidenced by induction of stemness-associated genes and self-renewal capacity. Targeting MUC1-C blocked induction of these stemness genes and inhibited self-renewal capacity, indicating that MUC1-C is necessary for the EBVaGC CSC state. By contrast, targeting EBNA1 increased (i) SOX2, KLF4 and NOTCH expression, and (ii) self-renewal capacity, indicating that EBNA1 suppresses the CSC state. These findings support a model in which EBNA co-opts MUC1-C to promote EBV latency and in turn cancer progression is dictated by a MUC1-C-dependent, EBNA1-independent pathway.

EBV has been linked to cancers of lymphoid and epithelial cell origin [2]. EBV-associated cancers have been attributed to prolonged latent EBV infection and chronic inflammation [11, 57, 63, 64]. The *MUC1* gene evolved in mammals to defend barrier tissues from viral infections and other biotic insults [13]. With infection and the resulting loss of homeostasis, activation of MUC1-C orchestrates inflammatory, proliferative and epigenetic remodeling responses necessary for tissue repair. The present work has uncovered a role for MUC1-C in linking latent EBV infection with EBVaGC progression. We demonstrate that MUC1-C regulates the expression of EBNA1, which is necessary for maintaining EBV latency [10]. MUC1-C induces EBNA1 transcripts by activating the EBV Q promoter. MUC1-C also forms a complex with EBNA1 and PLOD1, in support of regulating EBNA1 by transcriptional and post-translational mechanisms. In turn and in contrast to LMP1, which increases MUC1 expression [29], EBNA1 suppresses MUC1-C levels in an auto-regulatory pathway (Fig. 8A). In this manner, MUC1-C contributes to the upregulation of EBNA1 expression; whereas, EBNA1 has the capacity to fine-tune this pathway by controlling MUC1-C levels (Fig. 8A). These results identifying the intersection of EBNA1 and MUC1-C were of potential significance in that the role of EBNA1 in EBV viral latency as a mechanism directly involved in malignant transformation has remained controversial. We therefore reasoned that EBNA1 is exploiting MUC1-C to promote EBV latency and that the associated chronic inflammation contributes to prolonged MUC1-C activation and EBVaGC malignant progression (Fig. 8A).

Our results indicate that EBNA1 co-opts MUC1-C to drive pathways, such as DNA methylation, proliferation and survival, that contribute to EBV latency. For example, EBVaGC cells are notable for marked upregulation of DNMTs and DNA hypermethylation [11, 41, 42]. In studies of other types of cancer cells, MUC1-C induces DNMT expression and regulates global and TSG-specific DNA methylation patterns [15]. Our results in EBVaGC cells indicate that EBNA1 appropriates MUC1-C for upregulating DNMT expression (Fig. 8A). In support of those results, EBNA1 induces DNMT expression in YCCEL1 and SNU-719 cells by a MUC1-C-dependent mechanism. As further evidence, silencing MUC1-C in EBVaGC cells decreased DNMT expression and global DNA methylation. DNA hypermethylation in EBVaGC cells promotes the repression of host cell TSGs. Consistent with dependence on MUC1-C for DNA hypermethylation in EBVaGC cells and as selected examples, MUC1-C was necessary for repression of the *CDH1* and *RHOB* TSGs. These findings indicate that EBNA1 appropriates MUC1-C to drive DNMT expression and DNA methylation and thereby the regulation of host cell TSGs.

Host cell proliferation and survival are necessary for replication of the EBV genome. In EBVaGC cells, we found that MUC1-C drives expression of E2F1 and the E2F target gene signature. E2F1 is linked to p53 in regulating cell proliferation and fate [30, 49]. In driving EBV latency by pleotropic mechanisms, EBNA1 suppresses the p53 pathway to promote host cell proliferation and thereby EBV genome replication [10]. Our results in EBVaGC cells demonstrate involvement of MUC1-C in repressing p53 target genes. MUC1-C regulates p53 gene expression and the ARF/MDM2/p53 pathway [65, 66]. Furthermore, MUC1-C binds directly to p53 in the response to stress and regulates *CDKN1A* gene transcription [67]. Our findings demonstrate that EBNA1 diverts MUC1-C to repress p21 expression in YCCEL1 and SNU-719 cells. p53 also regulates the *BIRC5* gene encoding survivin to control apoptosis. EBNA1 upregulates survivin in B-lymphoma cells [68]. The present results demonstrate that EBNA1 exploits MUC1-C to drive survivin expression. Along these lines, clonogenic survival of YCCEL1 and SNU-719 cells was MUC1-C-dependent. These findings indicate that EBNA1 adopts MUC1-C for regulation of the p53 pathway in promoting proliferation and survival required for maintaining EBV latency (Fig. 8A).

EBV latency is sustained by EBNA1-mediated tethering of the EBV genome to cellular chromatin [38, 39]. The findings that (i) EBNA1 associates with MUC1-C, which localizes to chromatin [20, 21], and (ii) MUC1-C is necessary for tethering of EBNA1 to chromatin support the importance of EBNA1/MUC1-C complexes in integrating host cell and EBV gene expression. The interaction of MUC1-C and EBNA1 in regulating the expression of each other thus represents a mechanism for controlling the formation of EBNA1/MUC1-C complexes and thereby maintaining EBV latency (Fig. 8A). DNA methylation of the EBV genome in maintaining latency is also integrated with the regulation of MYC, CTCF, cohesin and PARP1 [11]. Our results demonstrate that MUC1-C is necessary for the regulation of MYC, which maintains EBV latency in B cells [27]. MUC1-C also regulates CTCF and RAD21 expression in EBVaGC cells, indicating that MUC1-C could contribute to latency by regulating 3-dimensional structures of the EBV genome. Consistent with the demonstration that MUC1-C regulates these multiple effectors of EBV latency, MUC1-C was necessary for (i) maintaining expression of EBV EBER1 and EBER2 latency genes, and (ii) suppressing the EBV BZLF1 and BMRF1 lytic phase genes (Fig. 8A). Arguably, these findings could be explained by EBNA1 dependency on MUC1-C for regulating expression of DNMTs, p21 and survivin, each of which contribute to latency.

EBV latency promotes a setting of chronic inflammation that contributes to cancer progression [11, 57, 63, 64]. MUC1-C is activated by viral infection, which in principle is reversible with resolution of the insult. As an alternative outcome, prolonged activation of MUC1-C by chronic inflammation drives epigenetic changes associated with inflammatory, proliferative and repair responses that become established in promoting cancer [13]. In this line of thinking for EBVaGC, MUC1-C was necessary for upregulation of the (i) SOX2 and KLF4 genes, which are linked to cancer progression [60, 61], and (ii) NOTCH genes that drive stemness [62] (Fig. 8B). Conversely, EBNA1 suppressed SOX2, KLF4 and NOTCH expression. Furthermore, MUC1-C and not EBNA1 was necessary for self-renewal capacity (Fig. 8B). In this regard, additional studies will be needed to determine if MUC1-C contributes to the regulation of EBV latency by driving stemness.

The mechanisms responsible for EBV latency and the association with cancer progression have largely remained unclear. The novelty of the present work resides in the demonstration that EBNA1 exploits key MUC1-C functions to promote EBV latency (Fig. 8A) and that, in response, chronic activation of MUC1-C drives EBVaGC malignant progression by EBNA1-independent pathways (Fig. 8B). In this regard, our findings unearth new insights into EBNA1-driven cancer pathogenesis. We note that the present work is limited to EBVaGC and may not extend to other EBV-driven cancers. This caveat notwithstanding, it is intriguing to speculate that MUC1-C may contribute to other cancers, such as nasopharyngeal carcinomas, linked to EBV infections. MUC1-C is expressed at the apical membranes of polarized epithelial cells and at markedly increased levels over the entire surface of cancer cells [13]. Moreover, activation of MUC1-C is associated with exposure of epitopes in the extracellular domain (ED) [13]. In this way, antibodies directed against conserved alpha-helices in the MUC1-C/ED have been generated for the development of (i) an allogeneic CAR T cell therapy, designated P-MUC1C-ALLO1, that has been well tolerated in the clinic, and (ii) an anti-MUC1-C (M1C) antibody-drug conjugate (ADC), which based on promising preclinical anti-cancer activity in the absence of toxicity is being advanced by the NCI NExT Program for IND-enabling studies and clinical evaluation. Further investigation will be needed to determine if these anti-MUC1-C agents under development are effective against EBV-associated cancers.

### Ethics approval and consent to participate

All methods were performed in accordance with the relevant guidelines and regulations. The IHC study was approved by Keio University (approval number: 20190057) with informed consent obtained from all participants. IHC images were derived from de-identified human tissue samples. The images do not contain information that can be used to identify individual participants. Written informed consent for publication of the images was therefore not applicable.

## Supplementary information

Supplementary Figure 1

Supplementary Figure 2

Supplementary Figure 3

Supplementary Figure 4

Supplementary Figure 5

Supplementary Figure 6

Supplementary Figure 7

Supplemental material

## Data Availability

The accession number for the RNA-seq data is GEO Submission GSE270984.

## Materials availability

Materials described here, including raw source data, will be made freely available to researchers for non-commercial purposes.
