# Supplementary figures and images for: MUC1-C auto-regulatory complex with EBNA1 is responsible for latent Epstein-Barr virus-associated gastric cancer progression
Source: Oncogene. 2025 Aug 5;44(38):3609–24. doi: 10.1038/s41388-025-03519-5 (PMC12436189; doi:10.1038/s41388-025-03519-5)

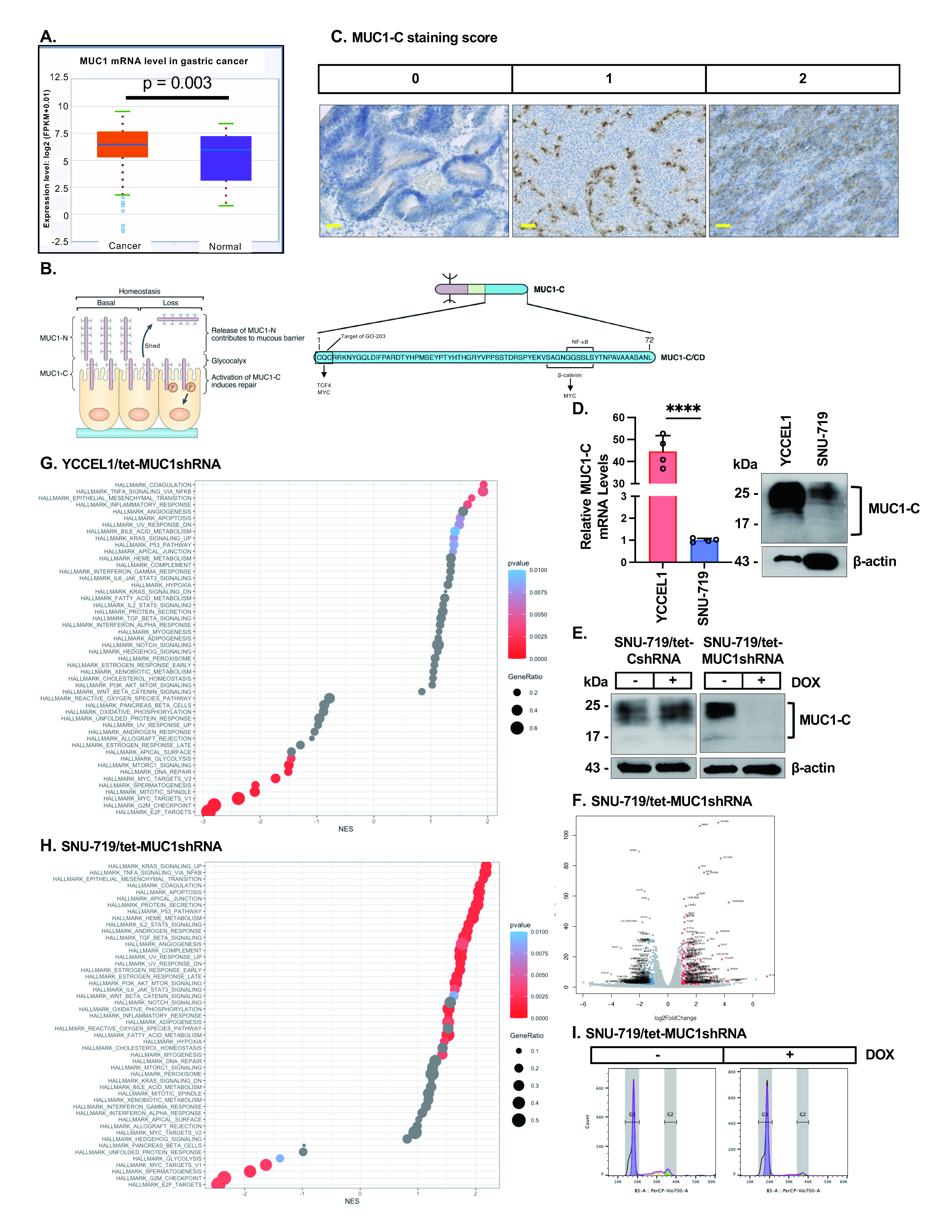

Supplement: Supplementary file 1 — Supplementary Figure 1 [file 41388_2025_3519_MOESM1_ESM.tif]

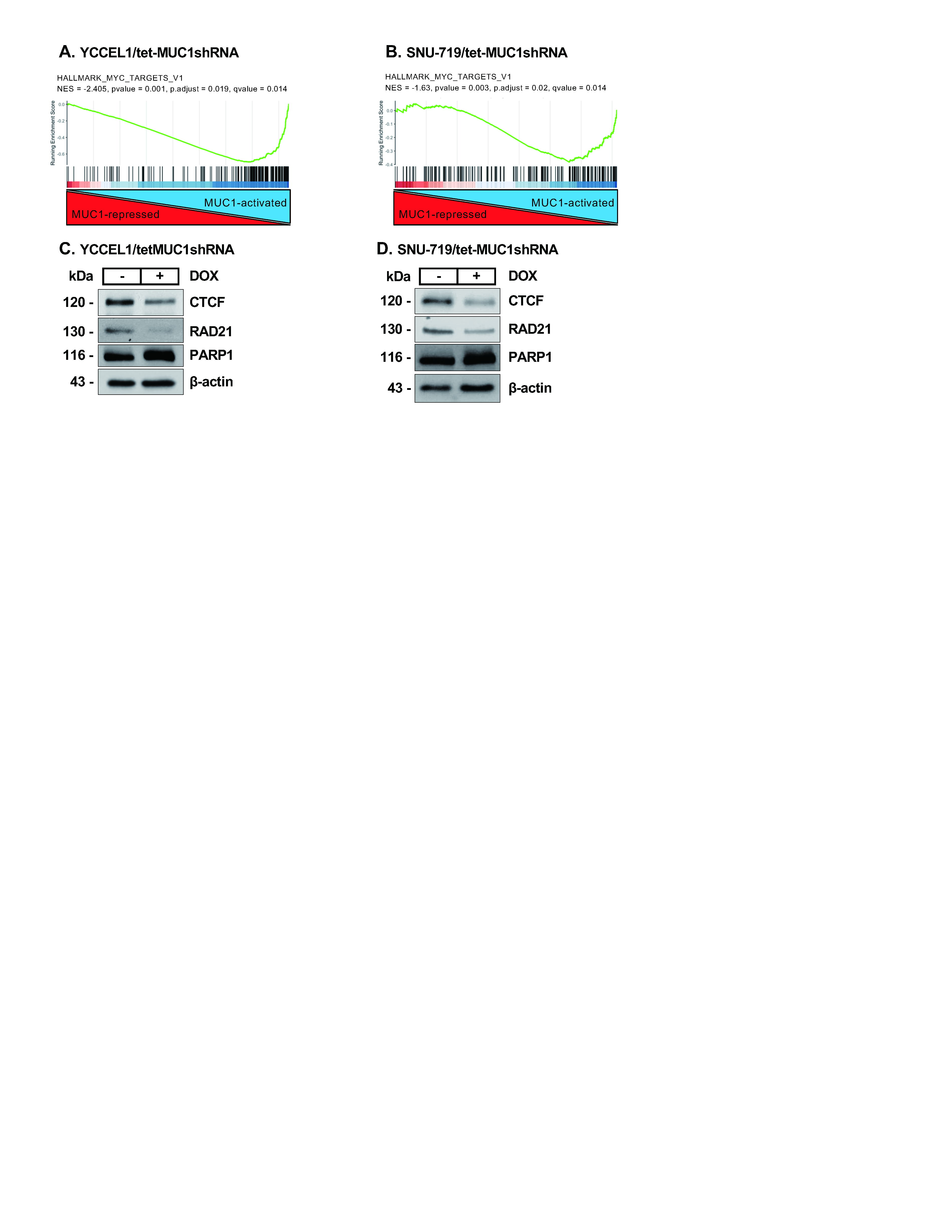

Supplement: Supplementary file 5 — Supplementary Figure 5 [file 41388_2025_3519_MOESM5_ESM.tif]

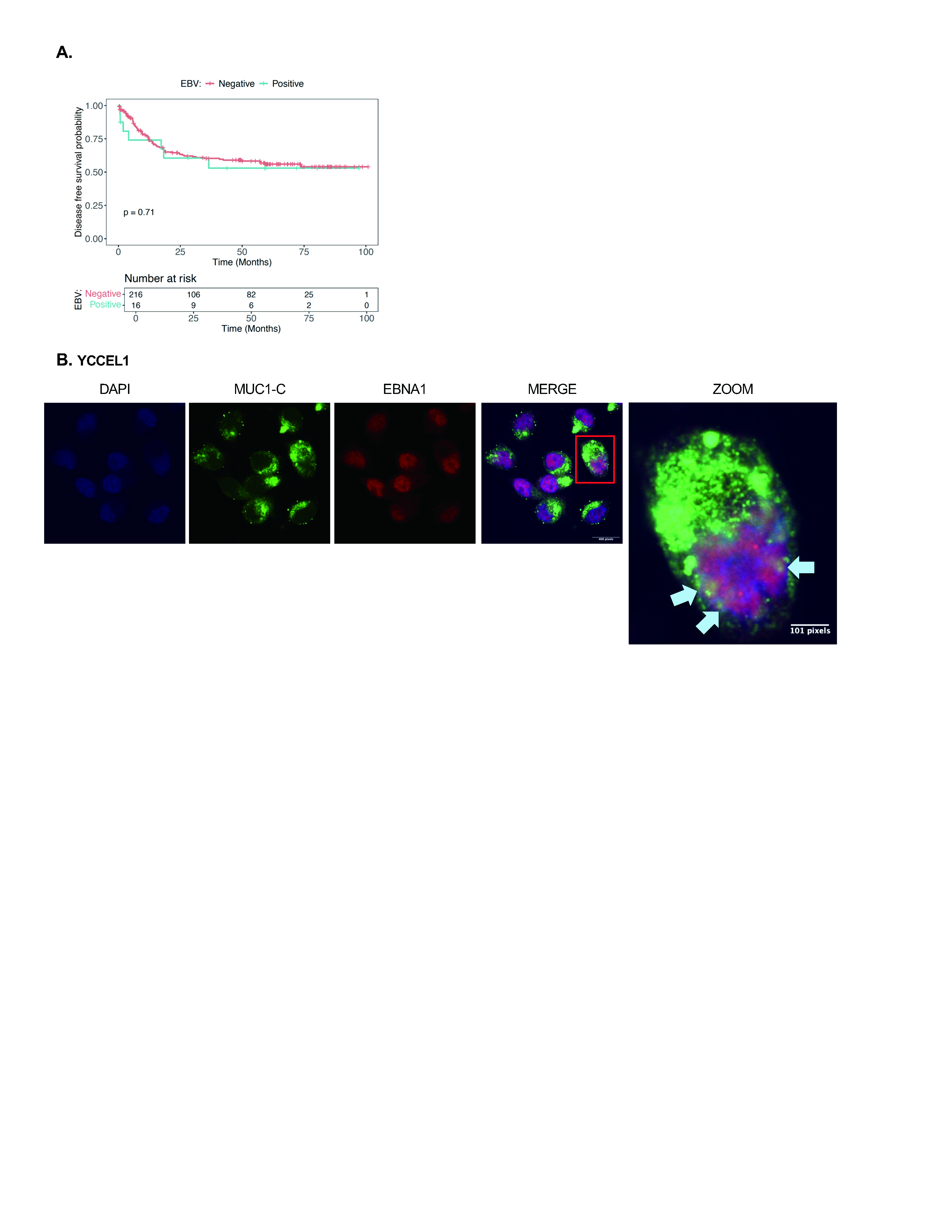

Supplement: Supplementary file 7 — Supplementary Figure 7 [file 41388_2025_3519_MOESM7_ESM.tif]
