# Supplementary material for: MUC1-C auto-regulatory complex with EBNA1 is responsible for latent Epstein-Barr virus-associated gastric cancer progression
Source: Oncogene. 2025 Aug 5;44(38):3609–24. doi: 10.1038/s41388-025-03519-5 (PMC12436189; doi:10.1038/s41388-025-03519-5)
Supplement: Supplementary file 8 — Supplemental material [file 41388_2025_3519_MOESM8_ESM.docx]

**SUPPLEMENTAL INFORMATION**

**Supplemental Figure Legends**

**Supplemental Figure S1. Effects of targeting MUC1-C in EBVaGC cells on gene expression. A.** Analysis of the ENCORI database for MUC1 expression in GC vs normal gastric tissues. **B.** MUC1 is expressed as a polypeptide that in the endoplasmic reticulum undergoes auto-cleavage at a conserved SEA domain. The resulting MUC1-N and MUC1-C subunits in turn form a non-covalent complex that is transported through the Golgi to the cell membrane. The MUC1-N/MUC1-C complex is disrupted by mechanical forces associated with loss of homeostasis, inducing release of MUC1-C from the cell surface and activation of MUC1-C for transducing intracellular stress signals. MUC1-C includes a 58 aa extracellular domain (ED), 28 aa transmembrane domain and 72 aa cytoplasmic domain (CD). The MUC1-C/ED is modified by N-glycosylation, resulting in expression of ~25 kDa glycoprotein. Unglycosylated MUC1-C is expressed as a 17 kDa protein. The intrinsically disordered MUC1-C/CD contains a CQC motif that binds directly to MYC and is a target for the GO-203 cell-penetrating peptide. MUC1-C/CD also binds directly to NF-κB. **C.** EBVaGC tumor samples from 15 patients who underwent surgical resections were analyzed by IHC for MUC1-C expression**.** Staining was scored as (i) negative or 0 in one (7%) tumor sample, (ii) intermediate in cell membranes or 1 in four (27%) tumor samples, and (iii) high in cell membranes and cytoplasm or 2 in 10 (67%) tumor samples. **D.** YCCEL1 and SNU-719 cells were analyzed for MUC1-C transcripts by qRT-PCR using primers listed in Supplemental Table S1. The results (mean±SD of four determinations) are expressed as relative levels compared to that obtained for SNU-719 cells (assigned a value of 1)**.** Lysates were immunoblotted with antibodies against the indicated proteins**.** The YCCEL1 cell lysate was diluted 10-fold relative to that used for SNU-719 cells. **E.** Lysates from SNU-719/tet-CshRNA and SNU-719/tet-MUC1shRNA cells treated with vehicle or DOX for 7 days were immunoblotted with antibodies against the indicated proteins. **F.** Volcano plot of down- and up-regulated genes in SNU-719/tet-MUC1shRNA cells treated with vehicle or DOX for 7 days. **G and H.** GSEA of RNA-seq data from YCCEL1 (**G**) and SNU-719 (**H**) cells with MUC1-C silencing using the indicated HALLMARK gene signatures. **I.** SNU-719/tet-MUC1shRNA cells were treated with vehicle or DOX for 7 days and analyzed for cell cycle distribution by flow cytometry.

**Supplemental Figure S2. Interaction of EBNA1 and MUC1-C in an auto-regulatory pathway. A.** SNU-719/tet-MUC1shRNA cells treated with vehicle or DOX for 7 days were analyzed for EBNA1 transcripts by qRT-PCR. The results (mean±SD of three determinations) are expressed as relative levels compared to that obtained for vehicle-treated cells (assigned a value of 1). **B.** Lysates from SNU-719 cells were precipitated with anti-MUC1-C or a control IgG. The precipitates and input lysate were immunoblotted with antibodies against the indicated proteins. **C.** YCCEL1/tet-MUC1shRNA cells treated with vehicle or DOX for 7 days were analyzed for PLOD1 transcripts by qRT-PCR. The results (mean±SD of four determinations) are expressed as relative levels compared to that obtained for vehicle-treated cells (assigned a value of 1). **D.** Lysates from YCCEL1/tet-MUC1shRNA cells treated with vehicle or DOX for 7 days were immunoblotted with antibodies against the indicated proteins (left). Lysates from YCCEL1/CshRNA and YCCEL1/MUC1shRNA#2 were immunoblotted with antibodies against the indicated proteins (right). **E.** Lysates from SNU-719/tet-MUC1shRNA cells treated with vehicle or DOX for 7 days were immunoblotted with antibodies against the indicated proteins. **F.** Lysates from SNU-719 cells expressing Flag-MUC1-CD were precipitated with anti-Flag or a control IgG. The precipitates and input lysate were immunoblotted with antibodies against the indicated proteins.

**Supplemental Figure S3. Effects of targeting EBNA1 and MUC1-C on DNMT expression. A.** Lysates from SNU-719 cells expressing an empty vector or EBNA1-DN were immunoblotted with antibodies against the indicated proteins. **B**. Chromatin from YCCEL1 and SNU-719 cells was immunoblotted with antibodies against the indicated proteins**.** **C.** Chromatin purified using method #1 (78840; Thermo Scientific), method #2 (ab117152; Abcam) and method #3 as described [1] were immunoblotted with antibodies against the indicated proteins. **D.** Chromatin from YCCEL1/CshRNA and YCCEL1/MUC1shRNA#2 cells was immunoblotted with antibodies against the indicated proteins**.** **E**. Chromatin from SNU-719/tet-MUC1shRNA cells treated with vehicle or DOX for 7 days was immunoblotted with antibodies against the indicated proteins. **F.** Lysates from SNU-719/tet-MUC1shRNA cells treated with vehicle or DOX for 7 days were immunoblotted with antibodies against the indicated proteins. **G.** Lysates from SNU-719 cells expressing the designated vectors were treated with vehicle or DOX for 7 days and immunoblotted with antibodies against the indicated proteins. **H.** Lysates from SNU-719 cells expressing an empty vector or EBNA1-DN were immunoblotted with antibodies against the indicated proteins. **I.** Lysates from SNU-719/tet-MUC1shRNA cells treated with vehicle or DOX for 7 days were immunoblotted with antibodies against the indicated proteins. **J and K.** YCCEL1/tet-MUC1shRNA (**J**) and SNU-719/tet-MUC1shRNA (**K**) cells were treated with vehicle or DOX for 7 days were analyzed for RHOB transcripts by qRT-PCR. The results (mean±SD of four determinations) are expressed as relative levels compared to that obtained for vehicle-treated cells (assigned a value of 1). **L.** YCCEL1 cells treated with vehicle or 0.5 μM DAC for 3 days were analyzed for RHOB transcripts by qRT-PCR. The results (mean±SD of four determinations) are expressed as relative levels compared to that obtained for vehicle-treated cells (assigned a value of 1).

**Supplemental Figure S4. MUC1-C regulates EBVaGC cell clonogenic survival. A.** GSEA of RNA-seq data from SNU-719 cells with MUC1-C silencing was performed using the HALLMARK P53 PATHWAY gene signature. **B and C.** SNU-719/tet-MUC1shRNA cells treated with vehicle or DOX for 7 days were analyzed for CDKN1A transcripts by qRT-PCR. The results (mean±SD of four determinations) are expressed as relative levels compared to that obtained for vehicle-treated cells (assigned a value of 1)(**B**)**.** Lysates were immunoblotted with antibodies against the indicated proteins (**C**)**.** **D.** YCCEL1/vector and YCCEL1/EBNA1-DN cells were analyzed for CDKN1A transcripts by qRT-PCR (left). The results (mean±SD of four determinations) are expressed as relative levels compared to that obtained for vehicle-treated cells (assigned a value of 1). Lysates were immunoblotted with antibodies against the indicated proteins (right). **E.** GSEA of RNA-seq data from SNU-719 cells with MUC1-C silencing was performed using the HALLMARK APOPTOSIS gene signature. **F and G.** SNU-719/tet-MUC1shRNA cells treated with vehicle or DOX for 7 days were analyzed for BIRC5/survivin transcripts by qRT-PCR. The results (mean±SD of four determinations) are expressed as relative levels compared to that obtained for vehicle-treated cells (assigned a value of 1) (**F**). Lysates were immunoblotted with antibodies against the indicated proteins (**G**)**. H and I.** Lysates from YCCEL1 (**H**) and SNU-719 (**I**) cells treated with vehicle or 3 μM GO-203 for 2 days were immunoblotted with antibodies against the indicated proteins. **J.** SNU-719/vector and SNU-719/EBNA1-DN cells were analyzed for colony formation**.** Shown are representative photomicrographs of stained colonies (left). The results (mean±SD of three determinations) are expressed as relative colony formation compared to that for vector cells (assigned a value of 1)(right). **K.** SNU-719 cells expressing the designated vectors were treated with vehicle or DOX for 7 days and analyzed for colony formation**.** Shown are representative photomicrographs of stained colonies (left). The results (mean±SD of three determinations) are expressed as relative colony formation compared to that for control cells (assigned a value of 1)(right). **L.** SNU-719 cells treated with vehicle or 5 μM GO-203 for 7 days were analyzed for colony formation**.** Shown are representative photomicrographs of stained colonies (left). The results (mean±SD of three determinations) are expressed as relative colony formation compared to that for vector cells (assigned a value of 1)(right).

**Supplemental Figure S5. MUC1-C regulates effectors of EBV latency in EBVaGC cells.** **A and B.** GSEA of RNA-seq data from YCCEL1 (**A**) and SNU-719 (**B**) cells with MUC1-C silencing was performed using the HALLMARK MYC TARGETS V1 gene signature. **C and D.** Lysates from YCCEL1/tet-MUC1shRNA (**C**) and SNU-719/tet-MUC1shRNA (**D**) cells treated with vehicle or DOX for 7 days were immunoblotted with antibodies against the indicated proteins.

**Supplemental Figure S6. MUC1-C is necessary for EBVaGC cell self-renewal capacity. A.** GSEA of RNA-seq data from SNU-719 cells with MUC1-C silencing was performed using the BENPORATH ES1 gene signature. **B.** Lysates from SNU-719/tet-MUC1shRNA cells treated with vehicle or DOX for 7 days were immunoblotted with antibodies against the indicated proteins. **C.** Lysates from YCCEL1/CshRNA and YCCEL1/MUC1shRNA#2 cells were immunoblotted with antibodies against the indicated proteins. **D.** SNU-719/tet-MUC1shRNA cells treated with vehicle or DOX for 7 days were analyzed for tumorsphere formation**.** Shown are representative photomicrographs of tumorspheres (left). The results (mean±SD of three determinations) are expressed as relative tumorsphere formation compared to that for vehicle-treated cells (assigned a value of 1)(right). **E.** SNU-719 cells treated with vehicle or 5 μM GO-203 for 7 days were analyzed for tumorsphere formation**.** Shown are representative photomicrographs of tumorspheres (left). The results (mean±SD of three determinations) are expressed as number of tumorsphers (right).

**Supplemental Figure S7. A.** Kaplan-Meier curves for DFS of patients with EBV-positive and -negative GCs in the ARCG cohort. **B.** IF staining of YCCEL1 cells for MUC1-C (green) and EBNA1 (red) expression. Nuclei were stained with DAPI. Arrows denote co-localization of EBNA1 and MUC1-C signals along the nuclear membrane.

**Supplemental Reference**

1 Méndez J, Stillman B. Chromatin association of human origin recognition complex, cdc6, and minichromosome maintenance proteins during the cell cycle: assembly of prereplication complexes in late mitosis. Mol Cell Biol 2000; 20: 8602-8612.

**Supplemental Tables**

**Supplemental Table S1. Primers used for qRT-PCR.**

| **Primer** | **FWD** | **REV** |
| --- | --- | --- |
| **MUC1-C** | **TACCGATCGTAGCCCCTATG** | **CTCACCAGCCCAAACAGG** |
| **EBNA1** | **GGTCGTGGACGTGGAGAAAA** | **GGTGGAGACCCGGATGATG** |
| **EBNA1 Qp** | **GTGCGCTACCGGATGGCG** | **ATGCCCTGAGACTACTCTCT** |
| **PLOD1** | **GGTCATTCTCTTCGCAGACAG** | **CCACCGGATACTTGGTCTCCA** |
| **DNMT1** | **AGAACGGTGCTCATGCTTACA** | **CTCTACGGGCTTCACTTCTTG** |
| **DNMT3a** | **AGTACGACGACGACGGCTA** | **CACACTCCACGCAAAAGCAC** |
| **DNMT3b** | **CCCAGCTCTTACCTTACCATCG** | **GGTCCCCTATTCCAAACTCCT** |
| **RHOB** | **CTGCTGATCGTGTTCAGTAAGG** | **TCAATGTCGGCCACATAGTTC** |
| **CDKN1A** | **AGGTGGACCTGGAGACTCTCAG** | **TCCTCTTGGAGAAGATCAGCCG** |
| **BIRC5** | **AGGACCACCGCATCTCTACAT** | **AAGTCTGGCTCGTTCTCAGTG** |
| **MYC** | **AGTAGAAATACGGCTGCACC** | **TTCGGGTAGTGGAAAACCAG** |
| **EBER1** | **TTTGCTAGGGAGGAGACGTGTGT** | **AAGCAGAGTCTGGGAAGACAACCA** |
| **EBER2** | **TTGCCCTAGTGGTTTCGGACACA** | **ACTTGCAAATGCTCTAGGCGGGAA** |
| **BZLF1** | **AGGCCAGCTAACTGCCTATC** | **TGATTCTGGGTTATGTCGGA** |
| **BMRF1** | **CGTGCCAATCTTGAGGTTTT** | **CGGAGGCGTGGTTAAATAAA** |
| **β-actin** | **GATGAGATTGGCATGGCTTT** | **CACCTTCACCGTTCCAGTTT** |

**Supplemental Table S2. Primers used for assessing EBV copy number.**

| **Primer** |  |
| --- | --- |
| **BALF5_F** | **5’-GAGCGATCTTGGCAATCTCT-3’** |
| **BALF5_R** | **5’-TGGTCATGGATCTGCTAAACC-3’** |
| **ACTBp_F** | **5’-AGAGCTACGAGCTGCCTGAC-3’** |
| **ACTBp_R** | **5’-AGCACTGTGTTGGCGTACAG-3’** |
